# Liver Imaging Reporting and Data System (LI-RADS): A Comprehensive Review

**DOI:** 10.7759/cureus.113706

**Published:** 2026-07-31

**Authors:** Tripti Prajapati, Rahul Dev, Udit Chauhan, Rahul Kumar

**Affiliations:** 1 Diagnostic and Interventional Radiology, All India Institute of Medical Sciences, Rishikesh, Rishikesh, IND; 2 Surgical Oncology, All India Institute of Medical Sciences, Rishikesh, Rishikesh, IND

**Keywords:** contrast-enhanced ultrasound, ct, hepatocellular carcinoma, li-rads, liver imaging reporting and data system, mri, ultrasound surveillance

## Abstract

Hepatocellular carcinoma (HCC) is a clinically significant primary liver malignancy that predominantly occurs in patients with cirrhosis and chronic liver disease. Early detection and accurate, non-invasive diagnosis are critical for improving survival outcomes, guiding therapeutic decision-making, and determining eligibility for liver transplantation. Imaging plays a central role in this process and frequently allows definitive diagnosis without the need for biopsy.

The Liver Imaging Reporting and Data System (LI-RADS), developed by the American College of Radiology (ACR), was created to standardize liver imaging acquisition, interpretation, categorization, and reporting in populations at risk for HCC. Since its introduction, LI-RADS has evolved to encompass ultrasound surveillance, contrast-enhanced ultrasound, multiphase computed tomography, magnetic resonance imaging, and treatment response assessment algorithms, including nonradiation and radiation-based therapies.

This narrative review provides a comprehensive overview of LI-RADS, encompassing its evolution and the latest developments in its diagnostic, surveillance, and treatment response algorithms.

## Introduction and background

Liver cancer ranks as the sixth most frequently diagnosed cancer globally and is the third leading cause of cancer-related deaths [1,2]. The majority of hepatocellular carcinoma (HCC) cases arise in the setting of chronic liver disease and cirrhosis, making regular surveillance essential for early detection. The Liver Imaging Reporting and Data System (LI-RADS) at-risk population includes patients with cirrhosis, chronic hepatitis B virus infection, and selected individuals with a prior history of HCC or liver transplantation, in whom standardized imaging plays a central role in surveillance, diagnosis, and treatment response assessment (TRA) [3,4]. Early diagnosis is crucial, as it directly influences access to potentially curative therapies such as surgical resection, liver transplantation, and local ablative treatments.

Surveillance aims to detect early-stage HCC in asymptomatic at-risk individuals, whereas diagnostic imaging is performed to characterize detected observations and determine the likelihood of HCC. Imaging has become central to the non-invasive diagnosis of HCC, with contrast-enhanced computed tomography (CT) and magnetic resonance imaging (MRI) serving as the primary diagnostic imaging modalities [3,4]. Prior to the adoption of standardized reporting systems, substantial variability existed in imaging techniques, terminology, and interpretation, leading to inconsistencies in diagnosis and management. To address these challenges, the American College of Radiology (ACR) developed the LI-RADS, which standardizes liver imaging through surveillance, diagnostic, and TRA algorithms, providing a common lexicon and standardized categories for lesion characterization and management.

Despite its widespread adoption, successful implementation of LI-RADS requires familiarity with evolving imaging criteria, technical recommendations, and periodic updates, which may present challenges in routine clinical practice. Although several comprehensive reviews have summarized LI-RADS, its evolution, and individual algorithms, the rapid evolution of the system, particularly the 2024 updates to the ultrasound (US) surveillance and CT/MRI and contrast-enhanced ultrasound (CEUS) TRA algorithms together with emerging post-update validation studies, has created a need for an updated, integrated review of the contemporary LI-RADS system. Accordingly, this narrative review provides an integrated review of the contemporary LI-RADS system by comprehensively reviewing all six LI-RADS algorithms, integrating the latest 2024 updates with emerging post-update validation evidence, and discussing their diagnostic performance, practical applications, current evidence, remaining challenges, and future directions.

## Review

Methodology

A structured literature search was conducted in PubMed using the keyword "LI-RADS" to identify publications relevant to the objectives of this narrative review. The search included articles published between 1 January 2011, corresponding to the introduction of LI-RADS, and 31 May 2026. Publication type filters available in PubMed were applied, including guideline documents, historical articles, introductory journal articles, review articles, scoping reviews, systematic reviews, meta-analyses, multicenter studies, observational studies, randomized controlled trials, validation studies, and research-supported publications. No Boolean operators or additional search filters were applied.

The search identified 411 records. Titles and abstracts were screened for relevance to the objectives of this review. Studies were considered eligible if they addressed the development, validation, diagnostic performance, surveillance, TRA, technical aspects, clinical applications, implementation, or updates of LI-RADS. Original research articles, multicenter studies, validation studies, systematic reviews, meta-analyses, guideline documents, and key historical publications relevant to the evolution of LI-RADS were eligible for inclusion. Particular emphasis was placed on studies evaluating LI-RADS across ultrasound, CEUS, CT/MRI, and TRA.

Studies were excluded if they were non-English publications, conference abstracts without full-text availability, editorials, commentaries, letters to the editor, duplicate publications, or articles lacking information relevant to the objectives of this review. Studies not primarily focused on LI-RADS, studies evaluating modified, institution-specific, or non-standard LI-RADS algorithms, studies evaluating only superseded LI-RADS versions without relevance to the current framework, and studies primarily addressing artificial intelligence, machine learning, deep learning, radiomics, prognostic or predictive models, pathology, biopsy, pediatric populations, transplantation, or other topics beyond the predefined scope of this review were also excluded.

**Figure 1:**
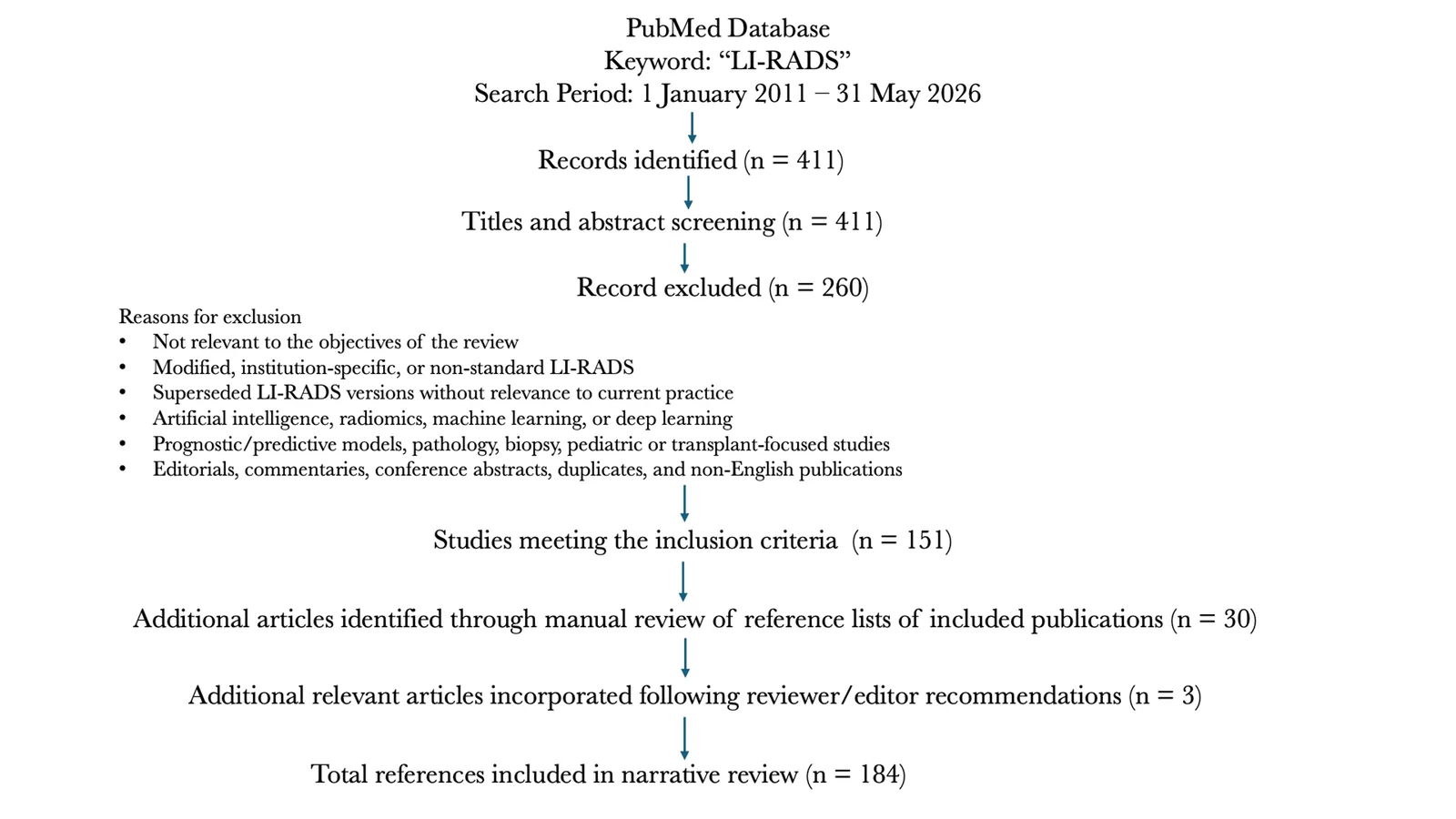
Literature selection flowchart. Abbreviations: LI-RADS, Liver Imaging Reporting and Data System.

Following title and abstract screening and full-text eligibility assessment, 151 articles met the inclusion criteria. An additional 30 relevant publications, including major guideline documents and key historical references, were identified through manual cross-referencing of the reference lists of the included studies. Subsequently, three additional publications were incorporated following reviewer and editor recommendations during manuscript revision. In total, 184 references were included in this narrative review. A flowchart summarizing the literature search, screening, eligibility assessment, and study selection process is presented in Figure 1.

Although a structured literature search and predefined selection criteria were employed to enhance transparency, this manuscript was conducted as a narrative review rather than a systematic review. Accordingly, full PRISMA reporting was not applied. No formal risk-of-bias assessment or quality appraisal of individual studies was performed. No original statistical analyses, meta-analysis, or meta-regression were undertaken. Reported summary estimates are derived from previously published studies and are presented descriptively.

Evolution of LI-RADS

The initial version of LI-RADS was introduced in 2011 when a small group of imaging experts met to address the need for greater consistency and clarity in liver imaging reports. The system was modeled after the Breast Imaging Reporting and Data System (BI-RADS) and was designed to categorize the probability of HCC [5]. Since its introduction, several updates have been released based on accumulating evidence and user feedback [5]. These updates include versions 2013.1, 2014, CEUS 2016, CT/MRI 2017, CEUS 2017 Essentials, and Ultrasound (US) Core 2017 [6,7]. Currently, the system is closely aligned with major hepatology guidelines, particularly those of the American Association for the Study of Liver Diseases (AASLD), and is intended exclusively for populations with a sufficiently high pre-test probability of HCC [3,8].

Over time, the scope of LI-RADS has expanded to include ultrasound surveillance, CEUS diagnostic algorithms, and structured TRA. Ultrasound is a favorable screening tool for identifying suspicious liver lesions in high-risk patients, whereas CEUS enhances lesion characterization and assists in the identification of HCC [9]. Despite this expansion, LI-RADS maintains a unified conceptual framework and standardized terminology across all imaging modalities, facilitating consistent communication among radiologists, hepatologists, surgeons, and oncologists.

**Figure 2:**
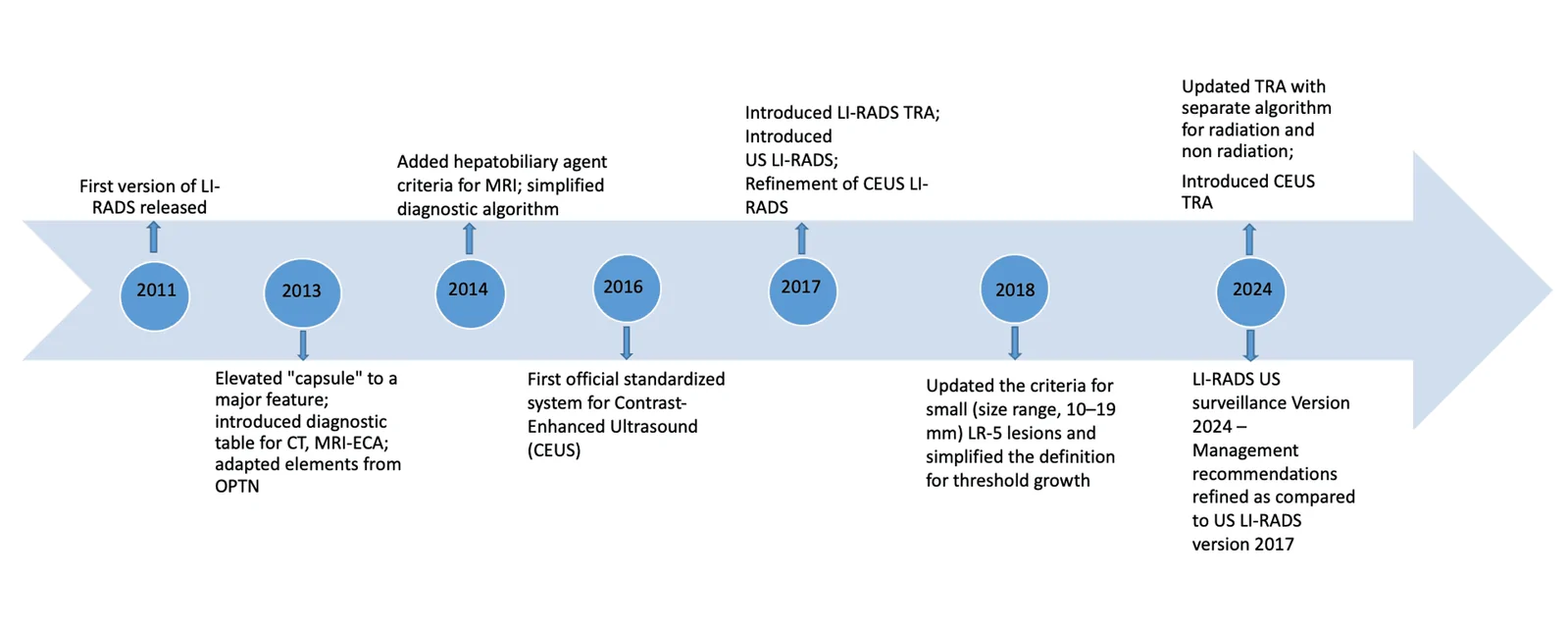
Key milestones in LI-RADS evolution. Created by the authors based on the American College of Radiology LI-RADS documents (2011-2024). Abbreviations: LI-RADS, Liver Imaging Reporting and Data System; CT, computed tomography; MRI, magnetic resonance imaging; ECA, extracellular contrast agent; CEUS, contrast-enhanced ultrasound; LR-5, definitely hepatocellular carcinoma; TRA, Liver Imaging Treatment Response Assessment; OPTN, Organ Procurement and Transplantation Network; TRA, treatment response assessment; US, ultrasound.

Currently, LI-RADS includes six algorithms: one for surveillance (US Surveillance v2024 Core), two for diagnosis (CT/MRI v2018 and CEUS v2017), and three for TRA (CT/MRI Nonradiation TRA v2024, CT/MRI Radiation TRA v2024, and CEUS Nonradiation TRA v2024) [10-15]. Figure 2 summarizes the major milestones in the development and evolution of LI-RADS.

Several recent comprehensive reviews have summarized the evolution of LI-RADS and its major algorithms. However, these reviews differ in their scope with respect to the algorithms covered, the incorporation of the latest updates, and the available validation evidence. Table 1 compares the principal characteristics of these reviews and highlights the distinguishing features of the present review before discussing the individual LI-RADS algorithms.

**Table 1:** Scope of previously published comprehensive LI-RADS reviews and the distinguishing contribution of the present review. Abbreviations: LI-RADS, Liver Imaging Reporting and Data System; HCC, hepatocellular carcinoma; US, ultrasound; CT, computed tomography; MRI, magnetic resonance imaging; CEUS, contrast-enhanced ultrasound; TRA, treatment response assessment.

Review	Primary focus	Scope of review	How the present review extends prior work
Elsayes et al., 2019 [5]	Conceptual and historical evolution of LI-RADS	Reviews the evolution of LI-RADS from its inception through v2018, including the US Surveillance, CT/MRI Diagnostic, CEUS Diagnostic, and CT/MRI Treatment Response algorithms, with future perspectives	Incorporates subsequent developments, including the 2024 US Surveillance and CT/MRI and CEUS Treatment Response Assessment updates, together with emerging post-update validation evidence
Chernyak et al., 2023 [6]	Evolution, current status, and future perspectives of LI-RADS	Comprehensive overview of all major LI-RADS algorithms, their evolution, remaining challenges, and future directions	Integrates the subsequent 2024 surveillance and treatment response updates with emerging validation studies while providing an updated synthesis of current evidence across all six algorithms
Chernyak, 2024 [16]	Role of LI-RADS in hepatocellular carcinoma	Reviews the role of LI-RADS in HCC surveillance, diagnosis, and clinical management	Broadens the scope by integrating all six algorithms, the latest guideline updates, and recently published validation evidence into a single review
Marks et al., 2021 [17]	Past, present, and future of LI-RADS	Reviews the origins, evolution, current state, and future objectives of LI-RADS, emphasizing its role in standardized liver imaging	Includes subsequent 2024 algorithm updates and summarizes the emerging evidence supporting their clinical implementation
Kierans et al., 2025 [18]	Recent updates, planned refinements, and future directions	Discusses recent LI-RADS refinements, planned updates, and future directions, with emphasis on the CT/MRI Diagnostic algorithm	Broadens the focus by providing a unified review of surveillance, diagnostic, and treatment response algorithms together with current validation evidence
Choi et al., 2024 [19]	Current status and future directions	Reviews the contemporary LI-RADS system, including the 2024 updates and future directions	Complements this overview by integrating emerging post-update validation studies and providing a comprehensive synthesis of all six algorithms within a single review
De Muzio et al., 2022 [20]	Narrative review of the LI-RADS algorithm	Reviews the contemporary US Surveillance, CEUS Diagnostic, and CT/MRI Diagnostic algorithms available at the time of publication	Updates the review by incorporating the latest surveillance and treatment response assessment algorithms together with subsequent validation evidence
Moura Cunha et al., 2021 [21]	CT/MRI LI-RADS	Comprehensive review of CT/MRI LI-RADS, including the contemporary CT/MRI Treatment Response algorithm available at the time of publication	Expands beyond CT/MRI by integrating US Surveillance, CEUS Diagnostic, and the updated CT/MRI and CEUS Treatment Response Assessment algorithms into a unified review
Present review	Integrated review of the contemporary LI-RADS system	Comprehensively reviews all six LI-RADS algorithms, including the latest surveillance, diagnostic, and treatment response assessment algorithms	Integrates the 2024 US Surveillance and CT/MRI and CEUS Treatment Response Assessment updates with emerging post-update validation evidence, summarizes current diagnostic performance and practical clinical applications, and discusses current evidence, remaining challenges, and future directions

Overview of all algorithms

According to the latest ACR LI-RADS updates, there are six LI-RADS algorithms [10-15]. A summary of these algorithms is provided in Table 2.

**Table 2:** LI-RADS algorithms. Abbreviations: LI-RADS, Liver Imaging Reporting and Data System; US, ultrasound; CEUS, contrast-enhanced ultrasound; CT, computed tomography; MRI, magnetic resonance imaging; HCC, hepatocellular carcinoma; TRA, treatment response assessment.

S. No.	LI-RADS algorithm	Indications & target population
1	US Surveillance Algorithm	For surveillance of HCC in cirrhotic and other high-risk patients, using unenhanced ultrasound
2	CEUS Diagnostic Algorithm	For diagnosis of HCC in cirrhotic and other high-risk patients, including liver transplant candidates and recipients posttransplant, using contrast-enhanced ultrasound (CEUS)
3	CT/MRI Diagnostic Algorithm	For diagnosis of HCC in cirrhotic and other high-risk patients, including liver transplant candidates and recipients post-transplant, using CT, MRI with extracellular agents (ECA), or MRI with hepatobiliary agents (HBA)
4	CEUS Nonradiation TRA Algorithm	For assessing response to nonradiation-based locoregional therapy (LRT) and surgical resection for HCC in cirrhotic and other high-risk patients, including liver transplant candidates and recipients post-transplant, using CEUS
5	CT/MRI Nonradiation TRA Algorithm	For assessing response to nonradiation-based locoregional therapy (LRT) and surgical resection for HCC in cirrhotic and other high-risk patients, including liver transplant candidates and recipients post-transplant, using CT, MRI with extracellular agents (ECA), or MRI with hepatobiliary agents (HBA)
6	CT/MRI Radiation TRA Algorithm	For assessing response to radiation-based LRT for HCC in cirrhotic and other high-risk patients, including liver transplant candidates and recipients post-transplant, using CT, MRI with extracellular agents (ECA), or MRI with hepatobiliary agents (HBA)

LI-RADS algorithms provide standardized criteria for category assignment to ensure clear communication. The US Surveillance algorithm applies to the entire examination and includes US-1 (negative), US-2 (subthreshold), and US-3 (positive) categories, along with visualization scores (VIS-A, VIS-B, VIS-C) to reflect image quality [10].

The CEUS and CT/MRI diagnostic algorithms share eight observation-level categories based on the likelihood of benignity or malignancy: LR-NC (not categorizable); LR-1 through LR-5 (definitely benign to definitely HCC); LR-M (malignant, not specific for HCC); and LR-TIV (tumor in vein) [16]. LR-5 carries a ≥95% positive predictive value for HCC in adults with cirrhosis (excluding vascular causes) or chronic hepatitis B. However, this diagnostic performance has been established only in patients meeting LI-RADS eligibility criteria and should not be extrapolated to individuals without established HCC risk factors, in whom diagnostic specificity may be substantially reduced. Management is category-specific: LR-3 typically warrants repeat or alternative imaging in three to six months, whereas LR-4 requires multidisciplinary discussion (MDD) to determine follow-up imaging, biopsy, or treatment [11,12].

The CT/MRI TRA algorithm further provides lesion-level assessment after nonradiation-based locoregional therapies, such as ablation, conventional transarterial chemoembolization (cTACE), and drug-eluting bead transarterial chemoembolization (DEB-TACE), and radiation-based therapies, such as stereotactic body radiotherapy (SBRT) or transarterial radioembolization (TARE), using LR-TR nonevaluable, nonviable, viable, nonprogressing, and equivocal categories [13,14]. The CEUS TRA algorithm was introduced in 2024 for assessment after nonradiation locoregional therapies and includes LR-TR nonevaluable, nonviable, viable, and equivocal categories [15].

Surveillance versus diagnostic imaging

LI-RADS clearly distinguishes between surveillance and diagnostic imaging based on clinical intent. Surveillance aims to detect HCC at an early, potentially curable stage and therefore prioritizes sensitivity, whereas diagnostic imaging prioritizes specificity to allow confident noninvasive diagnosis. Ultrasound serves as the primary modality for surveillance, while multiphase CT, MRI, and CEUS are used for diagnostic characterization of detected lesions [17].

LI-RADS ultrasound surveillance

Surveillance for HCC aims to detect tumors at an early, potentially curable stage, thereby reducing disease-related mortality. Its effectiveness depends on identifying populations at sufficient risk, using reliable and acceptable surveillance tests, and ensuring access to effective treatment. Current surveillance recommendations from the European Association for the Study of the Liver (EASL), the AASLD, and the Asian Pacific Association for the Study of the Liver (APASL) endorse ultrasound as the primary surveillance modality in high-risk individuals [3,22,23].

Ultrasound remains the most widely used surveillance technique because it is inexpensive, widely available, and free of ionizing radiation [24]. Nevertheless, its diagnostic performance may be limited by operator dependence, obesity, and heterogeneous liver parenchyma. To improve reporting consistency and communicate examination quality, LI-RADS incorporates standardized surveillance categories together with visualization scores that guide subsequent management. The LI-RADS US Surveillance algorithm, introduced in 2017 and updated in 2024, focuses on early lesion detection rather than definitive diagnosis and is aligned with the 2023 AASLD practice guidance for HCC surveillance [25].

Surveillance Population

The current recommended surveillance population includes adults with cirrhosis, specifically those with Child-Turcotte-Pugh (CTP) class A or B disease, regardless of the underlying etiology [26]. It is also advised for selected adults with chronic hepatitis B infection without cirrhosis, including men older than 40 years and women older than 50 years from endemic regions, individuals of African ancestry at younger ages, those with a family history of HCC, and patients in Western populations receiving antiviral therapy with a PAGE-B score of 10 or greater [26]. In addition, depending on geographic region and individual risk assessment, surveillance may be considered for selected adults without cirrhosis who have chronic hepatitis B, chronic hepatitis C, or metabolic dysfunction-associated steatotic liver disease/metabolic dysfunction-associated steatohepatitis (MASLD/MASH) [10,27,28].

Conversely, surveillance is generally not recommended for patients with CTP class C cirrhosis who are not transplant candidates or for individuals with severe comorbid illnesses limiting life expectancy to less than one to two years when this cannot be improved through transplantation or other disease-directed therapy [10,28].

Observation

Within LI-RADS terminology, an observation refers to any focal area that differs in appearance from the surrounding liver on imaging. Such observations may represent pseudolesions or true lesions, and true lesions may be benign or malignant, arising from hepatocellular or non-hepatocellular origins [25,29].

Surveillance Category and Visualization Score

Each surveillance examination requires systematic assessment of liver visualization, identification of focal observations, lesion measurement, and assignment of a US category. In the context of surveillance, sensitivity should be prioritized over specificity. Therefore, when uncertainty exists between two categories, the one indicating a higher level of suspicion should be assigned [10].

The system standardizes reporting by incorporating both a surveillance category, which guides management, and a visualization score (VIS), which reflects technical adequacy and expected sensitivity. Surveillance examinations are categorized as US-1 (negative), US-2 (subthreshold), or US-3 (positive), each associated with a specific management recommendation. US-1 (negative) indicates no sonographic evidence of HCC and includes examinations demonstrating no focal observation or only definitely benign findings; routine surveillance at six-month intervals is recommended. US-2 (subthreshold) is assigned when one or more observations smaller than 10 mm are detected that are not definitely benign. As such lesions may represent early malignancy but are below the threshold for definitive characterization, short-interval follow-up with repeat ultrasound in three to six months is advised. US-3 (positive) denotes findings that warrant further diagnostic evaluation, including observations 10 mm or larger that are not definitely benign, areas of parenchymal distortion suspicious for an underlying mass, or newly detected thrombus in the portal or hepatic veins. These findings should prompt multiphase contrast-enhanced imaging with CT, MRI, or CEUS [10].

In addition to the surveillance category, each examination is assigned a VIS to indicate the expected sensitivity of the study [30]. The US LI-RADS VIS is a reliable and reproducible measure for predicting the expected sensitivity of ultrasound surveillance in detecting focal liver observations [31]. VIS-A reflects no or minimal limitations, with near-complete visualization of a homogeneous or mildly heterogeneous liver and high confidence in lesion detection. VIS-B indicates moderate limitations that may reduce sensitivity for detecting small (<10 mm) observations, such as parenchymal heterogeneity, moderate beam attenuation, or incomplete visualization of portions of the liver or diaphragm; surveillance remains acceptable but should be interpreted with awareness of reduced sensitivity. VIS-C denotes severe limitations that substantially compromise examination sensitivity, including severe heterogeneity, marked attenuation or shadowing, or non-visualization of large portions of the liver or diaphragm. In such cases, alternative imaging modalities should be considered for HCC surveillance [10].

Studies have shown that suboptimal ultrasound visualization, including US LI-RADS VIS C, is more common in patients with cirrhosis, MASLD, and obesity, while clinically significant fibrosis or cirrhosis, advanced age, and larger spleen size further contribute to diagnostic limitations [32,33]. Alternative surveillance imaging modalities following VIS-C include full MRI, abbreviated MRI, or multiphase CT [34,35]. Currently, there is insufficient evidence to recommend one modality over another. Therefore, clinicians should use their clinical judgment to select the most appropriate alternative modality based on individual patient factors. Recently, increasing attention has been directed toward the use of abbreviated MRI protocols for patients who are suboptimal candidates for ultrasound, supported by emerging preliminary data [36-38]. Currently, three main abbreviated MRI approaches are under investigation: non-contrast abbreviated MRI, abbreviated MRI with both pre-contrast and post-contrast sequences, and hepatobiliary phase-focused abbreviated MRI. Evidence suggests that abbreviated-protocol screening MRI performs similarly to complete-protocol diagnostic MRI, indicating that the two approaches may be used interchangeably in appropriate clinical settings [39].

Alpha-fetoprotein (AFP) serves as a complementary surveillance tool, and diagnostic imaging is recommended when AFP is positive, regardless of ultrasound findings [40]. A positive AFP result is defined as AFP ≥ 20 ng/mL or doubling of AFP (or more) or increasing on two consecutive tests [10].

LI-RADS US Surveillance Version 2024: Comparison With US LI-RADS Version 2017

The LI-RADS US Surveillance v2024 algorithm introduces several refinements to surveillance management compared with the 2017 version. In accordance with the 2023 AASLD guidance, patients who do not meet the US-3 positive criteria but have elevated or rising AFP (AFP ≥20 ng/mL) are now recommended to undergo diagnostic CT or MRI. For examinations assigned a VIS-C visualization score, indicating severe limitations in liver visualization, repeat ultrasound within three months may be considered; if VIS-C persists on follow-up, alternative surveillance modalities such as abbreviated MRI or multiphase CT should be considered. Management of US-2 (subthreshold) observations has also been revised. Rather than continuing surveillance for up to two years as recommended in LI-RADS v2017, LI-RADS v2024 advises repeat ultrasound at three- to six-month intervals for two follow-up examinations. If the observation is no longer visualized or remains <10 mm after these examinations, it may be reclassified as US-1 (negative), allowing resumption of routine six-month surveillance [41]. Additional updates include the recommendation that surveillance ultrasound be performed preferentially in the outpatient setting and that cine sweeps be routinely acquired in addition to standard static images to improve examination quality and lesion detection [10,18,19,25].

Early evaluation of LI-RADS US Surveillance v2024 has provided evidence regarding the performance of its updated algorithm. In a retrospective analysis of 407 patients with cirrhosis who underwent semiannual surveillance US as part of a prospective trial, v2024 demonstrated higher sensitivity but lower specificity for HCC detection than v2017. Sensitivity was 64.3% for v2024 versus 39.3%-42.9% for v2017, whereas specificity decreased from 92.6%-92.9% with v2017 to 82.1%-82.3% with v2024. All seven HCCs identified by v2024 but not v2017 on consensus assessment had increasing AFP levels, supporting a potential contribution of AFP to the increased sensitivity. Among patients with negative initial v2024 surveillance examinations, an initial VIS-C score was the only independent predictor of repeat VIS-C on subsequent US [42].

LI-RADS Ultrasound Surveillance: Technical Recommendations

According to the ACR LI-RADS US Surveillance v2024, ultrasound surveillance for HCC should include a systematic evaluation of the liver, portal venous system, and biliary tree using grayscale and Doppler imaging. The examination should assess liver morphology, document portal vein patency, and detect and characterize focal liver observations. Any detected observation should be documented with orthogonal images, lesion measurements, anatomical location, and Doppler assessment, where appropriate, while associated findings such as splenomegaly and ascites should also be recorded. Standardized image acquisition, including representative images and cine sweeps of both hepatic lobes, is recommended to optimize examination quality and lesion detection. However, the imaging protocol may be adapted according to institutional practice and clinical indications [10,25,26,40]. Detailed technical recommendations and standard imaging views are summarized in Table 3.

**Table 3:** Recommended image acquisition and technical parameters for ultrasound surveillance. Source: Adapted from the American College of Radiology LI-RADS Ultrasound Surveillance v2024 Core [10].

Component	LI-RADS US Surveillance v2024 Recommendations
Liver assessment	Systematic grayscale evaluation of the entire liver with representative transverse and longitudinal images
Portal venous system	Document main portal vein patency with grayscale and color Doppler; optional color Doppler assessment of right and left portal veins and hepatic veins
Biliary system	Assess gallbladder and bile ducts; measure common bile duct when feasible
Focal observations	Orthogonal grayscale and color Doppler images, three-dimensional measurements, anatomical location, and relationship to adjacent structures
Additional findings	Record splenomegaly, ascites, and other relevant findings
Standard views	Both hepatic lobes, portal vein, hepatic veins, gallbladder, kidneys, diaphragm, liver margins
Optional views	Wide-field-of-view imaging, expanded Doppler evaluation, linear transducer for surface nodularity
Cine sweeps	Recommended longitudinal and transverse cine sweeps of both lobes; focused cine sweeps for lesions ≥1 cm

Diagnostic LI-RADS: General principles

Diagnostic LI-RADS algorithms are intended for characterization of focal liver observations detected during surveillance or incidentally identified in patients at high risk for HCC. In contrast to surveillance imaging, diagnostic LI-RADS prioritizes specificity to allow confident non-invasive diagnosis and to minimize false-positive interpretations that could lead to inappropriate treatment.

Diagnostic LI-RADS is applied exclusively in at-risk populations, including patients with cirrhosis, chronic hepatitis B infection, or prior HCC. Application outside these populations reduces diagnostic accuracy and increases false-positive interpretations [3]. Separate but conceptually aligned diagnostic algorithms exist for CEUS and CT/MRI [43]. Despite modality-specific differences, all diagnostic pathways rely on arterial phase hyperenhancement (APHE), lesion size, and enhancement dynamics.

Contrast-Enhanced Ultrasound (CEUS) Diagnostic LI-RADS

Overview, clinical applications, and diagnostic performance of CEUS Diagnostic LI-RADS: The CEUS LI-RADS diagnostic algorithm was introduced in 2016 and updated in 2017 to standardize the characterization of focal liver observations in patients at risk for HCC [44]. CEUS employs intravenously administered microbubble contrast agents that remain strictly intravascular, enabling real-time assessment of lesion vascularity without ionizing radiation. Ultrasound contrast agents are generally safe and well tolerated, with a safety profile comparable to or better than that of CT and MRI contrast media [45,46]. Continuous real-time arterial phase imaging eliminates timing errors frequently encountered with CT and MRI and allows precise assessment of APHE and washout characteristics [47].

Within the LI-RADS framework, CEUS serves a diagnostic rather than surveillance role and is applicable to patients at high risk for HCC, including those with cirrhosis, chronic hepatitis B infection, or current or prior HCC. It is particularly useful for characterization of nodules measuring ≥10 mm detected on surveillance ultrasound, evaluation of indeterminate CT or MRI observations, clarification of APHE when cross-sectional imaging is limited by suboptimal contrast timing, and differentiation of tumor thrombus from bland thrombus [44,48]. CEUS LI-RADS applies only to examinations performed using pure blood-pool contrast agents and should not be used with combined blood-pool and Kupffer-cell agents such as Sonazoid [12,46].

A meta-analysis reported pooled sensitivity and specificity of 0.72 and 0.92, respectively, for HCC diagnosis using CEUS LI-RADS [49]. Additional meta-analyses have shown high diagnostic accuracy of CEUS in differentiating malignant from benign focal liver lesions, with pooled sensitivity and specificity of 0.92 and 0.87, respectively [50]. The diagnostic performance of CEUS LI-RADS for noninvasive HCC diagnosis in high-risk populations has been consistently validated across multiple studies and systematic reviews [51-53]. For noninvasive HCC diagnosis, the CEUS LR-5 category demonstrates high specificity ranging from 93% to 95%, with pooled sensitivity of approximately 71% [54-56]. These diagnostic performance estimates should be interpreted within the intended LI-RADS target population, as application in patients without established HCC risk factors may reduce diagnostic specificity. Beyond HCC diagnosis, CEUS LI-RADS effectively differentiates HCC from non-HCC malignancies through the combined application of LR-5 and LR-M criteria [57-59].

In many clinical settings, CEUS is regarded as a key imaging modality for the evaluation of focal liver lesions, including HCC, owing to its favorable cost-effectiveness profile, real-time assessment of vascular enhancement, and absence of ionizing radiation [60]. CEUS and CT/MRI LI-RADS should be regarded as complementary rather than interchangeable modalities. CEUS excels in lesion-level vascular characterization and assessment of arterial enhancement patterns, whereas CT and MRI provide comprehensive liver staging, treatment planning, and detection of additional lesions. Studies have shown that CEUS can facilitate further characterization of indeterminate CT/MRI observations, particularly LR-3, LR-4, and LR-M lesions, and may improve diagnostic categorization when APHE is not confidently demonstrated on cross-sectional imaging [61-64]. CEUS is also valuable for guiding biopsy, selecting viable biopsy targets, monitoring enhancement changes over time, and distinguishing tumor thrombus from bland thrombus [12,57]. In a comparative study, Wang et al. demonstrated that CEUS achieved higher sensitivity than contrast-enhanced MRI for LI-RADS-based HCC diagnosis, while both modalities showed excellent interobserver agreement for the assessment of major imaging features [65].

The reproducibility of CEUS LI-RADS has also been well established. Multiple studies have reported substantial interobserver agreement for lesion categorization and major imaging features [66-70]. A prospective multicenter study demonstrated substantial inter-reader agreement for overall CEUS LI-RADS categorization, particularly for LR-5 and LR-M observations [70]. Although agreement between CEUS and contrast-enhanced CT (CECT) LI-RADS categories was only fair across modalities, CEUS LI-RADS demonstrated higher sensitivity for detecting hepatic malignancies, whereas CECT LI-RADS showed superior performance for distinguishing HCC from non-HCC malignancies [71]. Similarly, a meta-analysis reported substantial agreement for major CEUS LI-RADS features and category assignments, although agreement was comparatively lower for LR-M features [68].

CEUS LI-RADS has shown particular utility in the evaluation of small liver nodules. Diagnostic performance for lesions ≤20 mm appears comparable to that observed for larger lesions, and good interobserver agreement has been reported for nodules measuring ≤30 mm [69,72,73]. These findings support the reliable characterization of small hepatic observations in high-risk patients, facilitating appropriate clinical decision-making regarding further diagnostic work-up or timely therapeutic intervention.

Sonazoid and Kupffer-phase imaging - emerging evidence: Recent studies have explored the incorporation of Kupffer-phase imaging using Sonazoid (perfluorobutane microbubbles), a contrast agent approved in several countries, including Japan and South Korea. Unlike pure blood-pool agents, Sonazoid demonstrates prolonged hepatic enhancement due to uptake by Kupffer cells [44]. Several studies have evaluated modified CEUS LI-RADS algorithms incorporating Kupffer-phase findings. A recent meta-analysis reported that the Sonazoid-modified LR-5 category achieved acceptable sensitivity (0.77) but lower specificity (0.88) for HCC diagnosis, suggesting that Kupffer-phase defects may be more appropriately considered ancillary rather than major diagnostic features [74]. Other studies have shown that incorporation of Kupffer-phase findings improves sensitivity while maintaining high specificity [75-77]. Furthermore, perfluorobutane-enhanced CEUS has demonstrated diagnostic performance comparable to MRI for characterization of small HCCs, although gains in sensitivity may occur at the expense of modest reductions in specificity [78-80]. Although Sonazoid-based CEUS LI-RADS modifications have shown promising results in recent studies, they remain investigational and have not been incorporated into the current official ACR CEUS LI-RADS v2017.

CEUS LI-RADS technique and technical recommendations: Accurate application of CEUS LI-RADS requires adherence to the technical and imaging acquisition recommendations of the ACR CEUS LI-RADS v2017. These key technical considerations are summarized in Table 4 [12].

**Table 4:** CEUS LI-RADS technique and technical recommendations. Source: Adapted from the American College of Radiology CEUS LI-RADS Diagnostic Algorithm v2017 Core [12]. Abbreviations: CEUS, contrast-enhanced ultrasound; LI-RADS, Liver Imaging Reporting and Data System.

Component	Recommendations
Required systems and modes	Ultrasound scanner with contrast-specific imaging capability, including dual-screen display and timer. Follow contrast-specific instructions provided by the scanner manufacturer
Contrast agents	Current CEUS LI-RADS applies only to pure blood-pool contrast agents. It does not apply to combined blood-pool/Kupffer cell agents (e.g., Sonazoid)
Precontrast imaging	Identify the target nodule(s). Determine optimal patient position (supine, oblique, or left lateral decubitus), optimal scan plane (preferably longitudinal to minimize out-of-plane respiratory motion), and optimal breathing technique (quiet or suspended: neutral, inspiration, or expiration)
Arterial phase (AP)	Continuous imaging from contrast injection until peak arterial phase enhancement to capture arterial phase hyperenhancement (APHE), characterize enhancement pattern, and detect early washout
Portal venous phase (PVP) and late phase (LP)	Intermittent imaging every ~30 s to minimize microbubble destruction, continued until complete clearance of microbubbles (approximately 4-6 min), to detect late washout and assess its degree
Suggested imaging	Sweep the liver during PVP or LP to identify additional nodules, which may appear as focal hypoenhancing observations
Recording (recommended and optional)	Continuous cine loop from microbubble arrival through peak arterial phase hyperenhancement (APHE) is a minimum requirement. Optionally, cine recording may be extended up to 60 s post-injection. Static images should be recorded at 60 seconds and during each intermittent acquisition thereafter
Imaging parameters	Use a low mechanical index (MI < 0.3) to avoid microbubble destruction. Use default machine settings
Dual-screen imaging	Use B-mode imaging for guidance and place calipers on the lesion simultaneously on both screens to facilitate enhancement characterization
Timing	Start the timer at the beginning of the saline flush immediately after completion of contrast injection (time 0). Record the time (in seconds) at which washout is first observed
Injection technique	Use a ≥20-gauge intravenous catheter. Central venous lines or infusion ports may be used if safety and aseptic conditions are met. Inject contrast manually over 2-3 s with constant pressure, followed by a 5-10 mL saline flush at approximately 2 mL/s. Repeat injections may be performed as needed according to manufacturer guidelines. Do not exceed the maximum recommended contrast dose
Diameter measurement	Measure lesion size on pre-contrast B-mode imaging. Use the same imaging plane and modality as prior examinations when assessing interval growth

CEUS LI-RADS major imaging features, LR-M criteria, and tumor in vein: The CEUS LI-RADS diagnostic algorithm relies on standardized imaging terminology and major imaging features for lesion characterization. The two principal major imaging features are APHE and washout. APHE is defined as enhancement during the arterial phase that is unequivocally greater than that of the surrounding liver parenchyma. The enhancement may be diffuse or partial but should not demonstrate a rim-like or peripheral discontinuous globular pattern. The enhancing portion of the lesion must appear more echogenic than the adjacent liver during the arterial phase, although partial APHE may also occur [12].

Washout refers to a visually assessed, time-dependent reduction in lesion enhancement relative to the surrounding liver, resulting in hypoenhancement. Washout may begin during or after the arterial phase and can occur even in lesions that do not exhibit APHE. Both the timing and degree of washout are diagnostically important. Early washout (onset <60 seconds after contrast administration) and/or marked washout favor categorization as CEUS LR-M, whereas late (≥60 seconds) and mild washout is a major imaging feature favoring HCC [81]. Mild washout is characterized by reduced enhancement relative to the liver while retaining some internal enhancement; even if a lesion becomes completely hypoenhancing after two minutes, the washout remains classified as mild provided that some enhancement persists during the initial two-minute interval [12].

The imaging features favoring assignment to the CEUS LR-M category include rim APHE, early washout, and marked washout. Rim APHE represents a spatial pattern of arterial enhancement predominantly confined to the lesion periphery rather than involving the lesion diffusely [12]. Early washout is defined as washout occurring within 60 seconds of contrast injection and is a key feature suggesting non-HCC malignancy [12]. Marked washout describes pronounced hypoenhancement, often appearing “black” or punched out relative to the surrounding liver parenchyma, typically within approximately two minutes after contrast administration [12].

Tumor in vein (TIV) is defined as the unequivocal presence of enhancing soft tissue within a vein, regardless of whether a contiguous parenchymal mass is identified [12]. Differentiation from bland thrombus is based largely on enhancement timing. Early enhancement within the vein occurring approximately simultaneously with hepatic arterial opacification favors TIV, whereas delayed enhancement several seconds after arterial opacification suggests portal venous inflow within a partially occlusive or recanalized bland thrombus. Observations meeting these criteria should be categorized as CEUS LR-TIV, and the radiology report should specify the most likely underlying etiology, such as HCC, intrahepatic cholangiocarcinoma, or another malignancy [12].

CEUS LI-RADS ancillary imaging features: Ancillary features (AFs) in CEUS LI-RADS may be used to modify category assignment when appropriate but cannot independently establish a diagnosis of HCC. These features are classified as those favoring malignancy (not specific for HCC), favoring HCC in particular, and favoring benignity. Definite growth, defined as an unequivocal spontaneous increase in lesion size over time that cannot be attributed to measurement variability or technical differences, is considered an AF favoring malignancy but not specific for HCC [82].

AFs favoring HCC in particular include nodule-in-nodule architecture and mosaic architecture. Nodule-in-nodule architecture refers to the presence of a smaller internal nodule with imaging characteristics distinct from those of the surrounding larger nodule and is considered suggestive of HCC in the setting of cirrhosis [83]. Mosaic architecture describes a heterogeneous internal appearance composed of multiple randomly distributed nodules or compartments demonstrating variable imaging characteristics and is also commonly associated with HCC [83,84].

AFs favoring benignity include long-term size stability and size reduction. Size stability is defined as the absence of meaningful interval change in lesion dimensions on imaging examinations performed at least two years apart in the absence of treatment. Size reduction refers to a definite spontaneous decrease in lesion size that cannot be explained by measurement variability, technical differences between examinations, imaging artifacts, or resorption of hemorrhagic components. These features support a lower likelihood of malignancy and may justify downgrading the category by one level when appropriate [12].

CEUS diagnostic algorithm and categories: CEUS LI-RADS comprises eight diagnostic categories: CEUS LR-NC (not categorizable), CEUS LR-1 (definitely benign), CEUS LR-2 (probably benign), CEUS LR-3 (intermediate probability of malignancy), CEUS LR-4 (probably HCC), CEUS LR-5 (definitely HCC), CEUS LR-M (malignant but not specific for HCC), and CEUS LR-TIV [12,85]. Category assignment follows a structured stepwise algorithm designed to standardize lesion characterization in patients at risk for HCC.

The first step is assessment of technical adequacy. Observations that cannot be adequately evaluated because of image degradation or incomplete imaging acquisition are categorized as CEUS LR-NC. The lesion must then be visible on baseline grayscale ultrasound, as CEUS cannot characterize sonographically occult observations [44]. Evaluation subsequently focuses on the major imaging features of APHE and washout. Nonrim APHE favors HCC, whereas rim APHE is more suggestive of non-HCC malignancy. Similarly, both the onset and degree of washout are critical determinants of category assignment. Unlike CT/MRI LI-RADS, threshold growth is not considered a major imaging feature in CEUS LI-RADS [44,86].

In general, lesions demonstrating APHE with late and mild washout are categorized with progressively higher certainty for HCC, culminating in CEUS LR-5 for lesions measuring ≥10 mm. In contrast, observations exhibiting rim APHE, early washout, or marked washout are categorized as CEUS LR-M because these features favor non-HCC malignancy [44]. Assignment of LR-3, LR-4, and LR-5 categories is based on the presence of APHE, lesion size, and washout characteristics, as detailed in Table 5 [12].

**Table 5:** CEUS LI-RADS diagnostic criteria based on enhancement pattern, size, and washout. Source: Adapted from the American College of Radiology CEUS LI-RADS Diagnostic Algorithm v2017 Core [12]. Abbreviations: APHE, arterial phase hyperenhancement; LI-RADS, Liver Imaging Reporting and Data System; CEUS, contrast-enhanced ultrasound.

Arterial phase hyperenhancement (APHE)	Nodule size (mm)	Washout pattern	LI-RADS category
No APHE	< 20	No washout	CEUS LR-3
No APHE	< 20	Late and mild washout	CEUS LR-3
No APHE	≥ 20	No washout	CEUS LR-3
No APHE	≥ 20	Late and mild washout	CEUS LR-4
APHE present	< 10	No washout	CEUS LR-3
APHE present	< 10	Late and mild washout	CEUS LR-4
APHE present	≥ 10	No washout	CEUS LR-4
APHE present	≥ 10	Late and mild washout	CEUS LR-5

Role of ancillary features and tie-breaking rules: AFs may be applied at the radiologist’s discretion to modify category assignment but cannot independently establish a diagnosis of definite HCC. Features favoring malignancy include definite growth, whereas nodule-in-nodule architecture and mosaic architecture favor HCC in particular. Features favoring benignity include size stability for at least two years and spontaneous size reduction [87]. The presence of one or more AFs favoring malignancy permits upgrading the category by one level, up to a maximum of CEUS LR-4, whereas AFs favoring benignity allow downgrading by one category. AFs must never be used to upgrade an observation to CEUS LR-5, and if conflicting AFs are present, no category adjustment should be made [12,88]. Tie-breaking rules are applied whenever uncertainty exists between two adjacent categories. In such situations, the observation should be assigned to the category reflecting the lower level of diagnostic certainty. Similarly, suspected but non-definitive TIV should not be categorized as CEUS LR-TIV. The final step of the algorithm is assessment for TIV, with the unequivocal presence of enhancing soft tissue within a vein resulting in assignment of the CEUS LR-TIV category [12,44].

CEUS LR-1 and CEUS LR-2 observations: The ACR CEUS LI-RADS, similar to the CT/MRI LI-RADS, does not provide strict definitional criteria for LR-1 and LR-2 categories but instead offers representative examples for lesions that are definitely or probably benign.

CEUS LR-1 encompasses observations that are unequivocally benign based on characteristic imaging features. Typical examples include simple cysts, hemangiomas, and areas of hepatic fat deposition or sparing. Fat deposition or sparing typically appears as a nonmasslike, nonspherical region of altered echogenicity (either hyperechoic or hypoechoic) within the liver parenchyma, demonstrating isoenhancement relative to the surrounding liver in all contrast phases. These areas characteristically occur in predictable locations, most commonly adjacent to the gallbladder fossa or anterior to the right portal vein in segment IV [12].

CEUS LR-2 includes observations that are probably benign but lack the classic features required for definite benign categorization. Criteria include a distinct solid nodule smaller than 10 mm that demonstrates isoenhancement in all vascular phases. If an isoenhancing solid nodule measures 10 mm or greater, it should be categorized as CEUS LR-3. Additionally, a nonmasslike isoenhancing observation of any size that does not have the typical appearance or location of hepatic fat deposition or sparing should be assigned to CEUS LR-2. Lesions previously categorized as CEUS LR-3 that demonstrate interval size stability for two years or longer may also be downgraded to CEUS LR-2 [12].

Management based on CEUS LI-RADS category: Management recommendations based on CEUS LI-RADS category are stratified according to the level of diagnostic certainty and estimated probability of malignancy.

For CEUS LR-NC (not categorizable) observations, alternative diagnostic imaging with CECT or MRI should be performed within three months, or repeat CEUS may be considered within the same interval [12,85].

For CEUS LR-1 (definitely benign) observations, patients should return to routine surveillance with a recommended follow-up interval of six months. Similarly, CEUS LR-2 (probably benign) observations generally warrant return to routine surveillance at six months; alternatively, repeat CEUS within six months may be performed if clinically indicated [12,85].

CEUS LR-3 observations, which carry an intermediate probability of malignancy, should undergo repeat CEUS or alternative diagnostic imaging with CT or MRI within six months. MDD may be appropriate in selected cases to guide individualized management. For CEUS LR-4 (probably HCC) observations, MDD is recommended to establish consensus management. If neither biopsy nor definitive treatment is planned, repeat CEUS or alternative diagnostic imaging with CT or MRI should be performed within three months [12,85].

CEUS LR-5 (definitely HCC) establishes the diagnosis of HCC, and patients should proceed to MDD for treatment planning. CEUS LR-M observations, which are probably or definitely malignant but not specific for HCC, require MDD for consensus management. Subsequent steps may include alternative imaging, repeat imaging, biopsy, or initiation of treatment, depending on clinical context [12,85].

For CEUS LR-TIV (tumor in vein), MDD is mandatory. Further evaluation may include biopsy and correlation with serum biomarkers, with the goal of determining the underlying etiology of TIV, such as HCC, intrahepatic cholangiocarcinoma, or another malignancy [12,85].

In special situations where no observation is detected on precontrast ultrasound, management depends on the indication for CEUS. If CEUS was performed following a positive screening or surveillance ultrasound examination, the patient should return to routine surveillance. However, if CEUS was performed to further characterize a known CT or MRI LR-3, LR-4, or LR-M observation, alternative diagnostic imaging with CECT or MRI is recommended [12,85].

CT/MRI Diagnostic LI-RADS: Historical Development and Clinical Application

CT/MRI LI-RADS was initially introduced in 2011 and subsequently refined through updates in 2013, 2014, 2017, and 2018, incorporating revisions to major imaging features, AFs, and the criteria for TIV (LR-TIV) [89]. A major advancement occurred in 2018 when the AASLD adopted LI-RADS into its clinical practice guidance for HCC, reinforcing its role as the standard framework for noninvasive diagnosis and management [90,91]. This update expanded the LR-5 category to include all 10-19 mm observations demonstrating nonrim APHE with washout appearance, while eliminating the previously required "-us" and "-g" qualifiers [92,93]. The definition of threshold growth was also harmonized with Organ Procurement and Transplantation Network (OPTN) criteria, requiring a lesion size increase of at least 50% within six months or less. Subsequently, the 2023 OPTN revision aligned its Class 5 criteria with the LR-5 definition used in LI-RADS [94,95].

CT/MRI LI-RADS is intended exclusively for patients at increased risk of developing HCC, including individuals with cirrhosis, chronic hepatitis B virus infection, current or previous HCC, and adult liver transplant candidates or recipients [92]. Although non-cirrhotic hepatitis C virus infection is not included within the official LI-RADS target population, and LI-RADS is therefore not routinely intended for use in these patients, several studies have reported satisfactory diagnostic performance of LI-RADS v2018 in this setting [96,97]. Conversely, LI-RADS should not be applied in individuals younger than 18 years, patients without recognized HCC risk factors, or those with cirrhosis related to congenital hepatic fibrosis or vascular disorders such as hereditary hemorrhagic telangiectasia, Budd-Chiari syndrome, chronic portal vein occlusion, cardiac congestion, or diffuse nodular regenerative hyperplasia [29,98]. Likewise, LI-RADS categories are not intended for pathologically confirmed benign or malignant lesions of non-hepatocellular origin, including hemangiomas [11].

The CT/MRI LI-RADS algorithm is designed for interpretation of multiphasic CECT and MRI performed with either extracellular or hepatobiliary contrast agents. Meta-analytic evidence has demonstrated comparable proportions of HCC and overall malignancy across LI-RADS categories irrespective of whether CT, extracellular contrast-enhanced MRI, or gadoxetate-enhanced MRI is used [99]. Although the ACR does not recommend one modality over another, pooled evidence indicates that MRI provides significantly higher per-observation sensitivity for HCC detection than CT while maintaining similar specificity [100]. Likewise, multiple studies have demonstrated superior performance of gadoxetate-enhanced MRI compared with dynamic CT for categorization of LR-5, LR-TIV, and LR-M observations and for the diagnosis of HCC, with improved sensitivity and comparable specificity [101,102]. Extracellular contrast-enhanced MRI has likewise been shown to improve visualization of APHE and washout, particularly in hypervascular HCCs [103]. Furthermore, sequential gadoxetic acid-enhanced MRI following CECT has been reported to improve the sensitivity for detecting small HCCs without compromising diagnostic specificity [104].

CT/MRI LI-RADS - technical considerations and key diagnostic definitions: The ACR has established standardized technical recommendations for CT and MRI acquisition and has also provided precise definitions for contrast phases, major imaging features, LR-M observations, TIV (LR-TIV), and AFs used in CT/MRI LI-RADS. Together, these technical standards and imaging definitions provide a uniform framework for lesion characterization and categorization, promoting consistent interpretation and improving the noninvasive diagnosis of HCC and other hepatic malignancies in at-risk patients [11]. The recommended CT and MRI acquisition protocols are summarized in Tables 6-8 [11,105,106].

**Table 6:** CT technical recommendations. Source: Adapted from the American College of Radiology CT/MRI LI-RADS Diagnostic Table v2018 Core [11].

Component	Recommendations
Recommended equipment	Multi-detector computed tomography (MDCT) scanner with ≥ 8 detector rows
Required imaging phases	Arterial phase (late arterial phase strongly preferred), portal venous phase, delayed phase
Suggested (optional) imaging	Pre-contrast (non-contrast) images in patients with prior loco-regional treatment, multiplanar reformations (axial, coronal, sagittal)

**Table 7:** Recommendations for MRI with extracellular contrast agents or gadobenate dimeglumine. Source: Adapted from the American College of Radiology CT/MRI LI-RADS Diagnostic Table v2018 Core [11].

Component	Recommendations
Recommended equipment	• 1.5T or 3T
	• Torso phased-array coil
Required images	• Unenhanced T1-weighted in-phase (IP) and opposed-phase (OP) imaging
	• T2-weighted imaging (fat suppression per institutional preference)
	• Multiphase T1-weighted imaging including precontrast imaging, arterial phase (late arterial phase strongly preferred), portal venous phase, delayed phase
Suggested or optional images	• Diffusion-weighted imaging, subtraction imaging, multiplanar acquisition, 1- to 3-h hepatobiliary phase with gadobenate dimeglumine (same sequence type as for multiphase, may use higher flip angle to increase T1 contrast)

**Table 8:** Recommendations for MRI with gadoxetate disodium. Source: Adapted from the American College of Radiology CT/MRI LI-RADS Diagnostic Table v2018 Core [11].

Component	Recommendations
Recommended equipment	• 1.5T or 3T
	• Torso phased-array coil
Required images	• Unenhanced T1-weighted in-phase (IP) and opposed-phase (OP) imaging
	• T2-weighted imaging (fat suppression per institutional preference)
	• Multiphase T1-weighted imaging including precontrast imaging, arterial phase (late arterial phase strongly preferred), portal venous phase, transitional phase(2 to 5 min after injection), hepatobiliary phase (same sequence type as for earlier phases; may use a higher flip angle to increase T1 contrast)
Suggested or optional images	• Diffusion-weighted imaging, subtraction imaging, multiplanar acquisitions

The use of optional MRI techniques, including diffusion-weighted imaging, subtraction imaging, and delayed hepatobiliary-phase imaging with gadobenate dimeglumine, can further improve the detection and characterization of hepatic observations [107].

Both extracellular contrast-enhanced MRI and hepatobiliary contrast-enhanced MRI have demonstrated excellent diagnostic performance for HCC detection and surveillance [108,109]. Among hepatocyte-specific contrast agents, both gadobenate dimeglumine (Gd-BOPTA) and gadoxetate disodium (Gd-EOB-DTPA) enable accurate noninvasive diagnosis of HCC [110]. Comparative studies suggest that gadobenate-enhanced MRI may provide greater sensitivity for detecting HCC, particularly small lesions, than gadoxetate-enhanced MRI, regardless of whether only dynamic images or combined dynamic and hepatobiliary-phase images are evaluated [111]. In addition to its diagnostic accuracy, MRI LI-RADS demonstrates good reproducibility. A systematic review and meta-analysis by Kang et al. reported substantial overall inter-reader agreement for both major imaging features and final LI-RADS category assignment, despite variability among individual studies [112].

Definitions and imaging characteristics of contrast phases in LI-RADS: The ACR defines standardized contrast phases to ensure consistent interpretation of multiphasic CT and MRI examinations performed with extracellular and hepatobiliary contrast agents [11]. In LI-RADS, the arterial phase refers specifically to the hepatic arterial phase and is characterized by complete enhancement of the hepatic artery and its branches without enhancement of the hepatic veins through antegrade flow [11]. The early arterial phase demonstrates enhancement of the hepatic artery before portal venous opacification, whereas the late arterial phase shows enhancement of both the hepatic artery and portal vein and is the preferred phase for HCC detection because APHE is often most conspicuous during this period [11].

The extracellular phase represents the period during which hepatic enhancement reflects extracellular distribution of contrast material. With extracellular contrast agents (ECAs) or gadobenate dimeglumine, it includes both the portal venous and delayed phases, whereas with gadoxetate disodium, it is limited to the portal venous phase. The portal venous phase is characterized by complete portal venous enhancement, enhancement of the hepatic veins through antegrade flow, and peak enhancement of the liver parenchyma [11]. The delayed phase, acquired approximately two to five minutes after contrast administration, demonstrates persistent but reduced enhancement of both portal and hepatic veins together with decreasing hepatic parenchymal enhancement [11].

With hepatobiliary contrast agents, the transitional phase occurs between the extracellular and hepatobiliary phases and reflects simultaneous intracellular and extracellular contrast distribution, producing similar signal intensity within the liver parenchyma and vascular structures. This phase is typically acquired two to five minutes after administration of gadoxetate disodium but is generally not obtained with gadobenate dimeglumine [11]. The hepatobiliary phase is characterized by relative hyperintensity of the liver parenchyma compared with hepatic blood vessels together with biliary contrast excretion. It is usually acquired approximately 20 minutes after administration of gadoxetate disodium or one to three hours following gadobenate dimeglumine. A hepatobiliary phase examination is considered suboptimal when the liver parenchyma fails to demonstrate greater signal intensity than the hepatic blood vessels [11].

Major imaging features in LI-RADS: The major imaging features used for CT/MRI LI-RADS include nonrim APHE, nonperipheral washout, enhancing capsule, lesion size, and threshold growth. These imaging features form the basis of CT/MRI LI-RADS categorization and are used in combination with lesion size to estimate the likelihood of HCC [11].

Nonrim APHE is defined as enhancement during the arterial phase that is unequivocally greater than that of the surrounding liver parenchyma and involves all or part of the observation without demonstrating a peripheral rim pattern. The enhancing portion of the lesion should show clearly higher attenuation or signal intensity than the adjacent liver on arterial phase imaging [11,83,113,114].

Nonperipheral washout refers to a visually assessed temporal reduction in enhancement relative to the surrounding liver parenchyma, resulting in hypoenhancement during the extracellular phase [11,83,113,114]. When ECAs or gadobenate dimeglumine are used, washout may be evaluated during either the portal venous or delayed phase, whereas with gadoxetate disodium, it should be assessed only during the portal venous phase. Washout may be identified in enhancing observations irrespective of whether APHE is present [11].

An enhancing capsule is recognized as a smooth, sharply marginated peripheral rim surrounding most or all of an observation that is thicker or more conspicuous than the fibrotic tissue commonly observed around background nodules. It is typically best appreciated during the portal venous, delayed, or transitional phases [11,83,113,114].

Lesion size is measured as the maximum outer-edge-to-outer-edge diameter of the observation, including the capsule when present [11]. Measurements should be obtained using the imaging phase, sequence, and plane that most clearly delineate lesion margins. Arterial phase images and diffusion-weighted imaging should be avoided for measurement when alternative sequences better define lesion boundaries because arterial enhancement may overestimate lesion size and diffusion-weighted imaging may be affected by geometric distortion [11].

Threshold growth is defined as an increase in lesion size of at least 50% within a period of six months or less [11]. This feature should be applied only when the observation represents a true mass, perfusion-related pseudolesions have been excluded, and prior CT or MRI examinations of appropriate quality are available for comparison. Whenever possible, measurements should be performed using the same imaging phase, sequence, and plane across serial examinations to ensure consistency [11].

Tumor in vein (LR-TIV): Tumor in vein (LR-TIV) is assigned when unequivocal enhancing soft tissue is identified within a vein, irrespective of whether a contiguous hepatic mass is visible [11,115,116]. Certain findings may increase suspicion for tumor invasion but are insufficient for a definitive diagnosis in isolation. These include venous occlusion with irregular or indistinct vessel walls, restricted diffusion within an occluded vein, an occluded or poorly visualized vein contiguous with a malignant hepatic observation, and heterogeneous intravascular enhancement that cannot be explained by imaging artifact [106,116]. In such situations, careful evaluation should be performed to identify definite enhancing intravascular soft tissue before assigning the LR-TIV category.

Once LR-TIV has been established, the probable underlying etiology should be specified according to the associated hepatic observation [106]. TIV contiguous with a targetoid mass is categorized as LR-TIV, possibly due to non-HCC malignancy, whereas continuity with an LR-5 observation indicates LR-TIV, definitely due to HCC. In the absence of a definite contiguous mass or when only an indeterminate observation is present, the lesion is categorized as LR-TIV, probably due to HCC [106]. Meta-analytic evidence supports HCC as the predominant cause of LR-TIV, with pooled proportions of approximately 71% for HCC and 29% for non-HCC malignancies [117].

LR-TIV indicates definite tumor invasion of a vein and therefore has important prognostic and therapeutic implications. The presence of tumor in a vein influences tumor staging, liver transplantation eligibility, and treatment selection, necessitating multidisciplinary evaluation [11,106,116]. Management recommendations for LR-TIV observations are summarized in the management recommendations section.

LR-M category: The LR-M category is assigned to hepatic observations demonstrating imaging features suggestive of malignancy that are not specific for HCC [11,106,118]. This category includes targetoid masses as well as non-targetoid lesions exhibiting one or more imaging features favoring non-HCC malignancy, including an infiltrative growth pattern, marked diffusion restriction, extensive necrosis or ischemia, or other imaging findings considered by the interpreting radiologist to be more consistent with a non-HCC malignant process.

Targetoid lesions are characterized by concentric internal zones that produce a target-like appearance and are identified in observations that do not satisfy LR-5 criteria and do not demonstrate TIV [11,119,120]. This morphology reflects peripheral tumor hypercellularity surrounding central fibrosis, ischemia, or necrosis and is most frequently encountered in intrahepatic cholangiocarcinoma, combined hepatocellular-cholangiocarcinoma, and other primary hepatic malignancies, although it may occasionally occur in atypical HCC [11]. Consequently, a targetoid appearance suggests, but does not establish, a diagnosis of non-HCC malignancy [11,119,120].

Characteristic targetoid enhancement includes rim APHE, peripheral washout, and delayed central enhancement [11,120]. Additional targetoid findings include peripheral diffusion restriction and a concentric pattern of hypointensity during the transitional or hepatobiliary phases, with greater hypointensity at the lesion periphery than centrally [11,120]. In hepatic observations measuring ≤3 cm, rim APHE, delayed central enhancement, targetoid diffusion restriction, and targetoid transitional/hepatobiliary phase appearance have all been independently associated with non-HCC malignancy [121].

Although LR-M observations most commonly represent intrahepatic cholangiocarcinoma or combined hepatocellular-cholangiocarcinoma, atypical HCC remains an important differential diagnosis [122]. Other considerations include metastatic disease, less common primary hepatic malignancies, and selected benign entities such as sclerosing hemangioma or hepatic abscess [11]. Whenever feasible, the most likely underlying diagnosis should be reported because it may influence decisions regarding biopsy, liver transplantation, and treatment planning. In selected patients, serum AFP and international normalized ratio (INR) may provide additional information supporting the noninvasive diagnosis of HCC [123]. Imaging findings including satellite nodules, T2-weighted hyperintensity, restricted diffusion, and the absence of capsule appearance in lesions demonstrating peripheral progressive enhancement favor the diagnosis of combined hepatocellular-cholangiocarcinoma [124].

Although LI-RADS v2018 demonstrates high specificity for distinguishing HCC from non-HCC primary liver malignancies, its sensitivity remains relatively limited [125]. Meta-analyses have consistently shown that most LR-M observations represent non-HCC malignancies; however, approximately 28-29% are ultimately diagnosed as HCC [126,127].

As LR-M observations indicate probable malignancy but are not specific for HCC, histopathological confirmation is frequently required before definitive treatment planning, particularly when distinguishing HCC from intrahepatic cholangiocarcinoma or combined hepatocellular-cholangiocarcinoma would alter clinical management [11,122-124]. Management recommendations for LR-M observations are summarized in the management recommendations section.

Ancillary features: AFs provide complementary imaging findings that refine the likelihood of benignity or malignancy beyond the major imaging features. Although they may increase diagnostic confidence and allow modification of the LI-RADS category, they should be applied at the discretion of the interpreting radiologist and must not be used to upgrade an observation to LR-5 [11]. AFs are grouped into three categories: those favoring malignancy in general, those favoring HCC in particular, and those favoring benignity. These features are summarized in Tables 9-11.

**Table 9:** Ancillary features favoring malignancy, not hepatocellular carcinoma in particular. Source: Adapted from the American College of Radiology CT/MRI LI-RADS Diagnostic Table v2018 Core [11]. Abbreviations: CT, computed tomography; MRI, magnetic resonance imaging.

Feature	Definition
Ultrasound visibility as a discrete nodule	Identification of a discrete nodule or mass on unenhanced ultrasound that corresponds to an observation detected on CT or MRI
Subthreshold growth	Unequivocal increase in lesion size that does not meet criteria for threshold growth
Corona enhancement	Perilesional enhancement observed in the late arterial or early portal venous phase, attributed to venous drainage from the tumor into surrounding liver parenchyma
Fat sparing in a solid mass	Relative absence of fat within a solid lesion compared with a steatotic background liver, or relative fat deficiency within an inner nodule compared with a steatotic outer nodule
Restricted diffusion	Signal intensity on diffusion-weighted imaging that is unequivocally higher than liver parenchyma and/or apparent diffusion coefficient (ADC) values that are unequivocally lower than liver, not attributable solely to T2 shine-through effect
Mild-to-moderate T2 hyperintensity	Signal intensity on T2-weighted imaging that is mildly or moderately greater than liver parenchyma and similar to or less than that of a non-iron-overloaded spleen
Iron sparing in a solid mass	Relative absence of iron within a solid lesion compared with an iron-overloaded background liver, or relative iron deficiency within an inner nodule compared with a siderotic outer nodule
Transitional phase hypointensity	Signal intensity during the transitional phase that is unequivocally lower, in whole or in part, than the surrounding liver parenchyma
Hepatobiliary phase hypointensity	Signal intensity during the hepatobiliary phase that is unequivocally lower, in whole or in part, than the surrounding liver parenchyma

**Table 10:** Ancillary features favoring hepatocellular carcinoma in particular. Source: Adapted from the American College of Radiology CT/MRI LI-RADS Diagnostic Table v2018 Core [11].

Feature	Definition
Non-enhancing capsule	A capsule-like peripheral border surrounding an observation that is visible but does not demonstrate contrast enhancement
Nodule-in-nodule architecture	Presence of a smaller inner nodule within a larger nodule, with the inner component demonstrating imaging characteristics that differ from the surrounding outer nodule
Mosaic architecture	Internal composition characterized by multiple randomly distributed compartments or nodules within a mass, often displaying variable signal intensity or enhancement patterns
Fat in mass greater than adjacent liver	Presence of intralesional fat that is greater, in whole or in part, than the fat content of the surrounding liver parenchyma
Blood products in mass	Evidence of intralesional or perilesional hemorrhage in the absence of recent biopsy, trauma, or therapeutic intervention

**Table 11:** Ancillary features favoring benignity. Source: Adapted from the American College of Radiology CT/MRI LI-RADS Diagnostic Table v2018 Core [11]. Abbreviations: T2WI, T2-weighted imaging.

Feature	Definition
Size stability ≥ 2 years	No significant change in observation size measured on exams ≥ 2 years apart in the absence of treatment
Size reduction	Unequivocal spontaneous decrease in size over time, not attributable to artifact, measurement error, technique differences, or resorption of blood products
Parallels blood pool enhancement	Temporal pattern in which enhancement eventually reaches and then matches that of blood pool
Undistorted vessels	Vessels traversing an observation without displacement, deformation, or other alteration
Iron in mass, more than liver	Excess iron in a mass relative to background liver
Marked T2 hyperintensity	Intensity on T2WI markedly higher than liver and similar to bile ducts or other fluid-filled structures
Hepatobiliary phase isointensity	Intensity in hepatobiliary phase nearly identical to liver

In CT/MRI LI-RADS, the use of AFs in conjunction with major imaging features has been shown to improve the sensitivity of LI-RADS while maintaining high specificity for the diagnosis of HCC [128]. AFs can be evaluated across CT, MRI with ECAs, MRI with hepatobiliary agents (HBAs), and ultrasound, thereby providing additional information for lesion characterization. Compositional features, including fat sparing within solid masses, excess fat relative to the adjacent liver, and intralesional or perilesional blood products, are partially evaluable on CT and fully evaluable on both MRI techniques. In contrast, restricted diffusion, mild-to-moderate T2 hyperintensity, and iron sparing are MRI-specific findings, whereas transitional phase hypointensity and hepatobiliary phase hypointensity are exclusive to hepatobiliary contrast-enhanced MRI, highlighting its added value for lesion characterization [11,83].

AFs favoring HCC include nonenhancing capsule appearance, nodule-in-nodule architecture, mosaic architecture, fat in mass, and blood products in mass, most of which can be identified on both CT and MRI, although their detection may vary depending on the imaging modality and technique [82]. AFs favoring malignancy include ultrasound visibility as a discrete nodule, subthreshold growth, corona enhancement, fat sparing in a solid mass, restricted diffusion, mild-to-moderate T2 hyperintensity, iron sparing in a solid mass, transitional phase hypointensity, and hepatobiliary phase hypointensity, whereas size stability ≥2 years, size reduction, parallel blood pool enhancement, undistorted vessels, iron in mass (greater than the liver), and marked T2 hyperintensity are considered supportive of benignity [129-131]. Furthermore, all AFs favoring malignancy in general (except fat sparing in a solid mass) and those favoring HCC in particular (except blood products in a mass) have been individually associated with HCC or malignancy [132].

Among the AFs favoring HCC in particular, a nonenhancing capsule and mosaic architecture demonstrate high specificity, and their application has been shown to improve the sensitivity of HCC diagnosis [133]. Likewise, among AFs favoring malignancy, mild-to-moderate T2 hyperintensity, restricted diffusion, transitional phase hypointensity, and hepatobiliary phase hypointensity are the strongest predictors of HCC [82,134-137]. Of these, hepatobiliary phase hypointensity is the most sensitive AF for the diagnosis of HCC [135]. Inter-reader agreement is generally higher for AFs favoring malignancy than for those favoring benignity [138]. However, although selected AFs can improve sensitivity, particularly when used in conjunction with major imaging features, their incremental contribution to overall diagnostic performance remains relatively modest [139].

CT/MRI LI-RADS diagnostic algorithm and categorization: The CT/MRI LI-RADS diagnostic algorithm follows a structured stepwise approach for the evaluation of focal liver observations in patients at high risk for HCC [119]. The process begins with detection of a focal observation and measurement of its maximum diameter, followed by assessment of major imaging features, including nonrim APHE, nonperipheral washout, enhancing capsule, and threshold growth [24,140]. Lesion categorization is subsequently determined according to lesion size and the number of major imaging features present [20,29,141]. In parallel, all examinations should be evaluated for TIV (LR-TIV), which is established by the presence of unequivocal enhancing soft tissue within a vein and indicates definite tumoral vascular invasion.

The CT/MRI LI-RADS system comprises eight diagnostic categories ranging from definitely benign observations (LR-1) to definitely HCC (LR-5), as well as categories for non-HCC malignancy (LR-M), TIV (LR-TIV), and non-categorizable examinations (LR-NC) [11]. The diagnostic categories and their clinical significance are summarized in Table 12.

**Table 12:** CT/MRI LI-RADS diagnostic categories. Source: Adapted from the American College of Radiology CT/MRI LI-RADS Diagnostic Table v2018 Core [11]. Abbreviations: CT, computed tomography; MRI, magnetic resonance imaging; LI-RADS, Liver Imaging Reporting and Data System; HCC, hepatocellular carcinoma; ICC, intrahepatic cholangiocarcinoma.

CT/MRI LI-RADS category	Diagnostic interpretation	Clinical meaning
LR-NC	Not categorizable	Inadequate examination due to image omission or degradation
LR-1	Definitely benign	No malignancy
LR-2	Probably benign	Very low likelihood of malignancy
LR-3	Intermediate probability	Indeterminate likelihood of malignancy
LR-4	Probably HCC	High likelihood of hepatocellular carcinoma
LR-5	Definitely HCC	Diagnostic of hepatocellular carcinoma
LR-M	Probably or definitely malignant, not specific for HCC	Suggests non-HCC malignancy (e.g., ICC and metastasis)
LR-TIV	Tumor in vein	Definite vascular invasion by tumor

**Figure 3:**
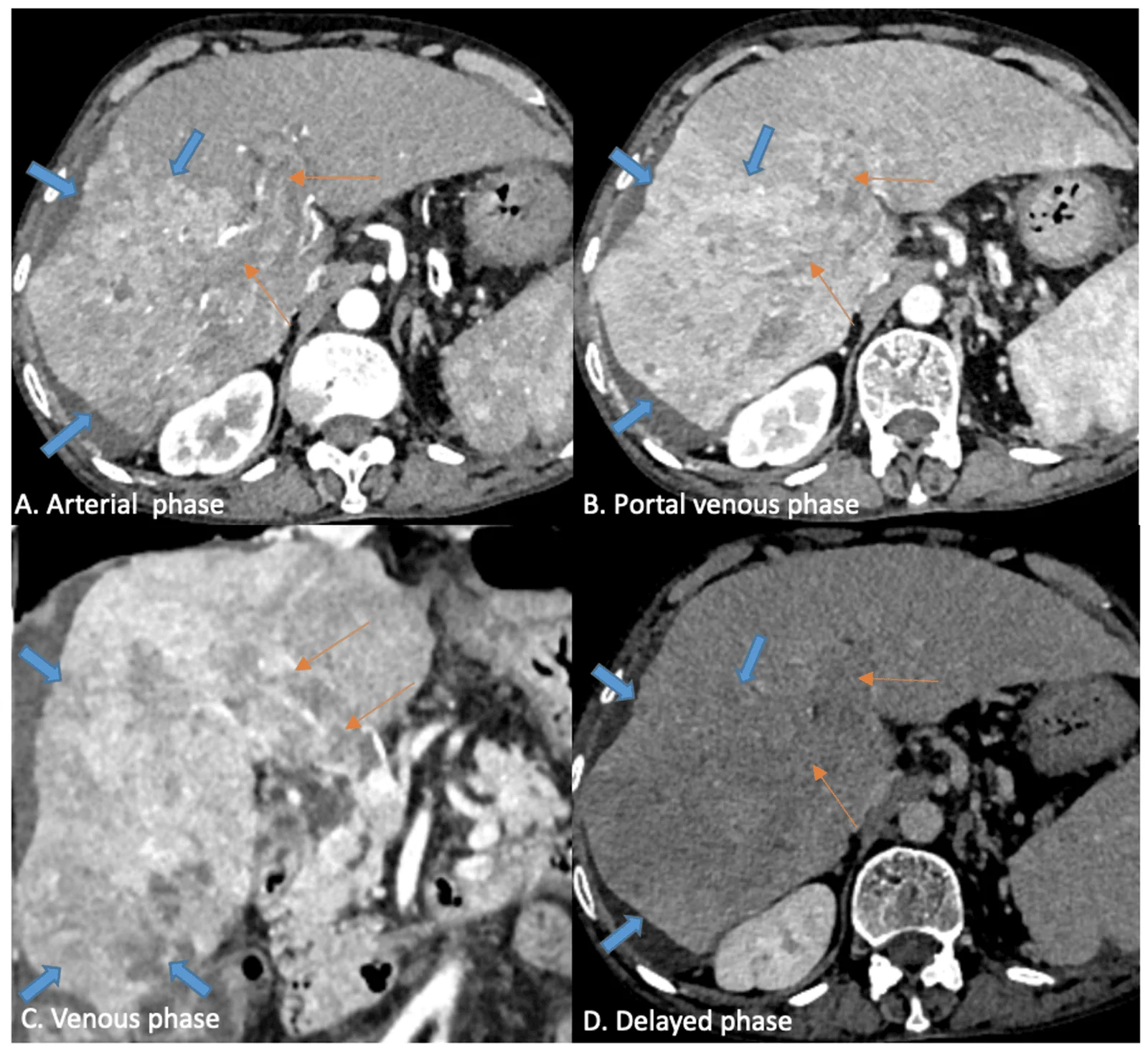
LR-TIV with LR-5 observation on multiphasic contrast-enhanced CT. Multiphasic contrast-enhanced CT images obtained during the arterial (A), portal venous (B), venous (C), and delayed (D) phases, including axial images (A, B, and D) and a coronal reconstruction (C), demonstrate enhancing soft tissue within the main, right, and left portal veins (orange arrows), consistent with LR-TIV. The tumor in the right portal vein is contiguous with a large hepatic observation in the right lobe (blue arrows), demonstrating nonrim arterial phase hyperenhancement, nonperipheral washout on the portal venous (B), venous (C), and delayed (D) phases, and imaging features consistent with an LR-5 observation in a patient with chronic liver disease.

During categorization, observations demonstrating definitive benign features are assigned LR-1, whereas probably benign lesions are categorized as LR-2. LR-NC is reserved for examinations that cannot be adequately interpreted because of missing images or significant image degradation. LR-M is assigned to observations exhibiting imaging features suggestive of malignancy but not specific for HCC, such as targetoid morphology, while LR-TIV is assigned whenever unequivocal enhancing soft tissue is identified within a vein, irrespective of whether a contiguous hepatic mass is visible [11]. A representative example illustrating the imaging appearance of TIV (LR-TIV) on CT is shown in Figure 3.

**Figure 4:**
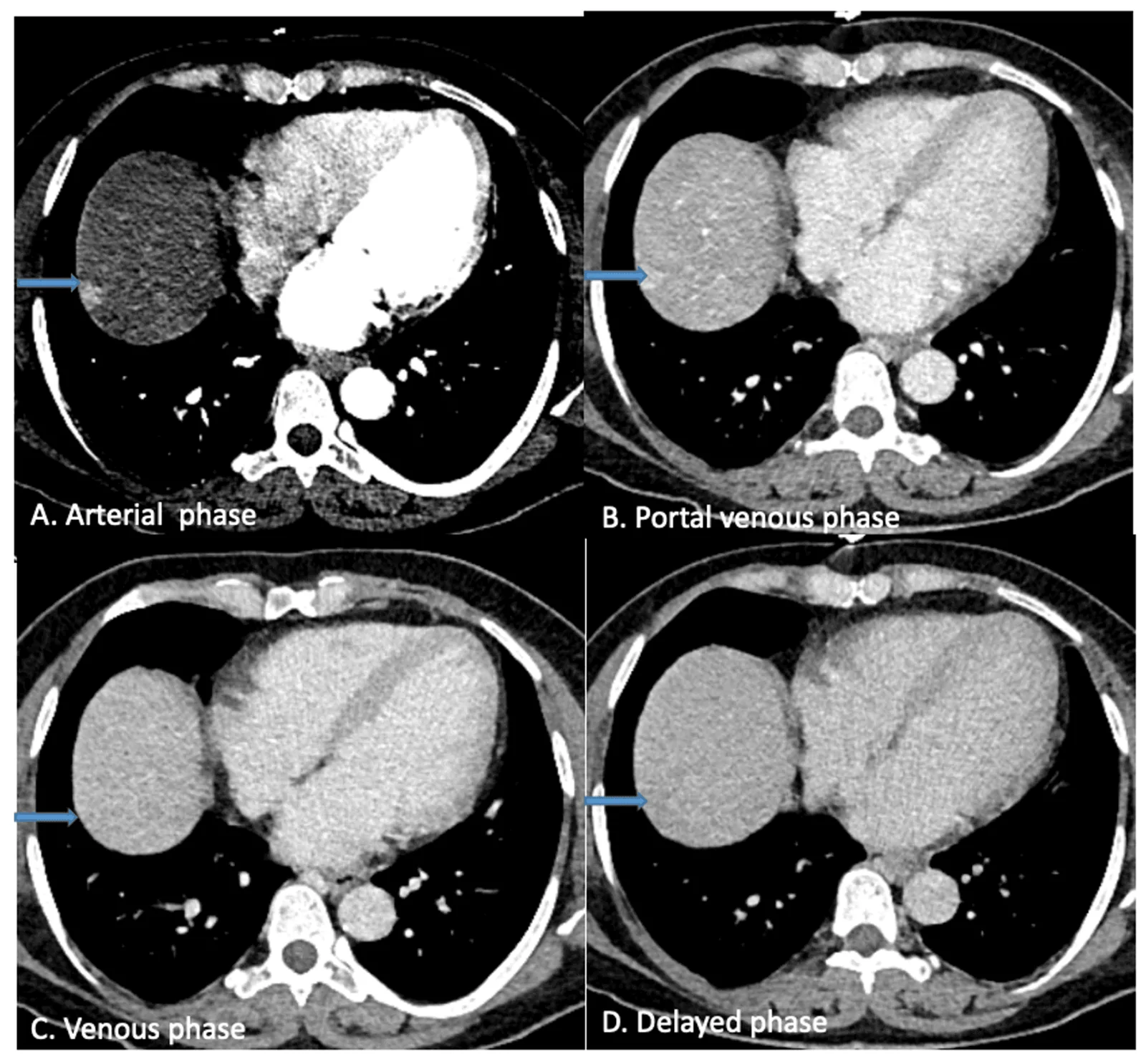
LR-3 observation on multiphasic contrast-enhanced CT. Axial multiphasic contrast-enhanced CT images obtained during the arterial (A), portal venous (B), venous (C), and delayed (D) phases demonstrate a well-defined hepatic observation measuring <10 mm (blue arrow) with nonrim arterial phase hyperenhancement. The observation demonstrates persistent enhancement on the portal venous phase and isoenhancement on the subsequent venous and delayed phases, without additional major or ancillary imaging features. In a patient with chronic liver disease, these imaging findings are consistent with an LR-3 observation according to the Liver Imaging Reporting and Data System (LI-RADS).

**Figure 5:**
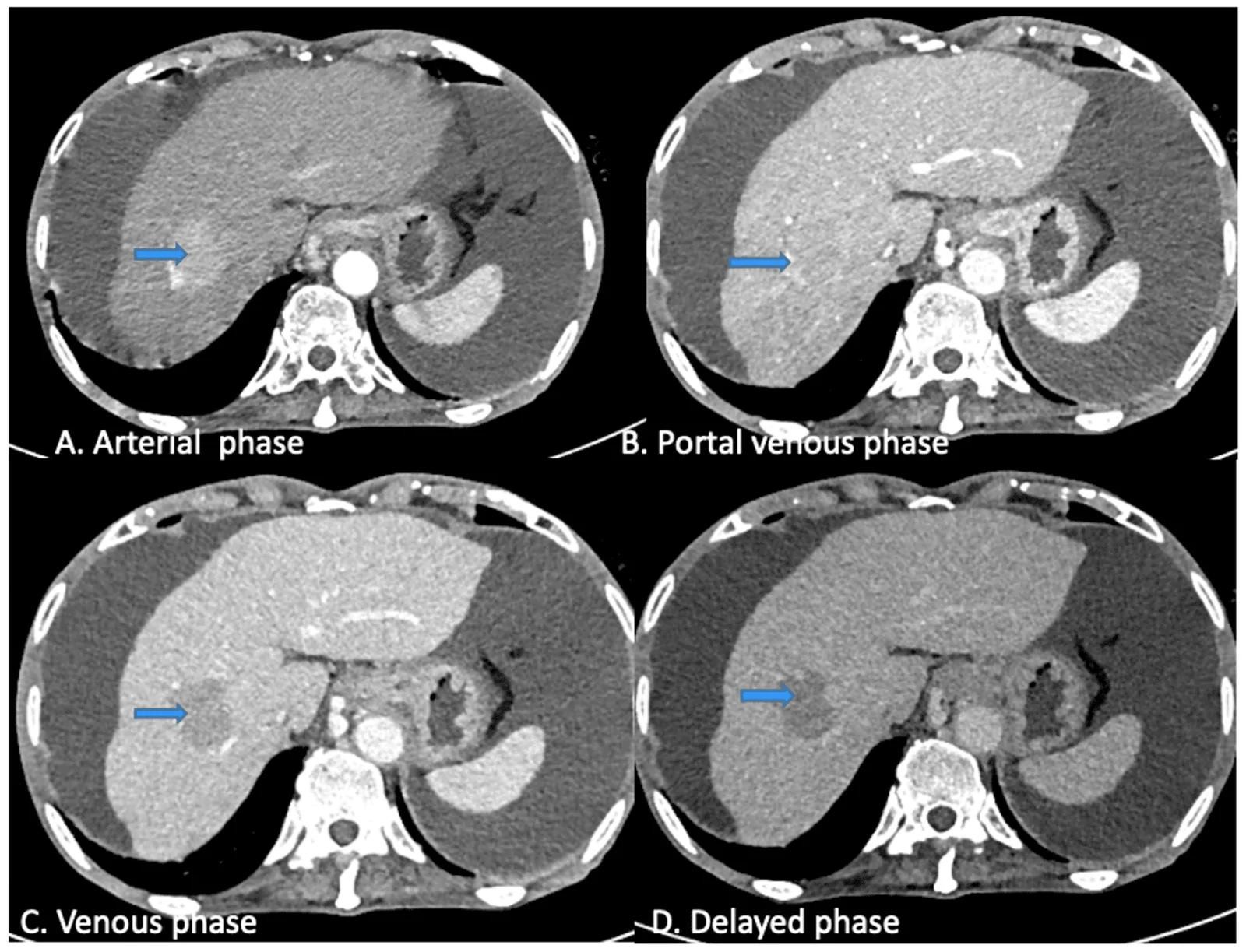
LR-5 observation on multiphasic contrast-enhanced CT. Axial multiphasic contrast-enhanced CT images obtained during the arterial (A), portal venous (B), venous (C), and delayed (D) phases demonstrate a well-defined hepatic observation measuring >2 cm (blue arrow). The observation demonstrates nonrim arterial phase hyperenhancement, nonperipheral washout on the portal venous, venous, and delayed phases, and an enhancing capsule on the delayed phase. Background findings include chronic liver disease with ascites. These major imaging features are consistent with an LR-5 observation (definitely hepatocellular carcinoma according to the Liver Imaging Reporting and Data System (LI-RADS)).

LR-3, LR-4, and LR-5 categorization: For all remaining untreated observations, categorization is primarily determined by lesion size, the presence or absence of nonrim APHE, and the number of additional major imaging features [20,141]. The categorization criteria for LR-3, LR-4, and LR-5 are summarized in Table 13 [11]. In general, the likelihood of HCC increases with increasing lesion size and accumulation of major imaging features. Representative CT appearances of LR-3 and LR-5 observations are shown in Figure 4 and Figure 5, respectively.

**Table 13:** CT/MRI LI-RADS categorization of LR-3, LR-4, and LR-5 observations. Source: Adapted from the American College of Radiology CT/MRI LI-RADS Diagnostic Table v2018 Core [11]. Abbreviations: APHE, arterial phase hyperenhancement; LI-RADS, Liver Imaging Reporting and Data System.

Nonrim APHE	Observation size	Additional major features	LI-RADS category
Absent	<20 mm	Any number	LR-3
	≥20 mm	None	LR-3
		One or more	LR-4
Present	<10 mm	None	LR-3
		One or more	LR-4
	10-19 mm	None	LR-3
		Enhancing capsule only	LR-4
		Nonperipheral washout or threshold growth	LR-5
		Two or more additional major features	LR-5
	≥20 mm	None	LR-4
		One or more	LR-5

Although the likelihood of HCC increases progressively from LR-3 to LR-5, these categories should not be considered equivalent. LR-3 observations remain indeterminate and generally require interval follow-up or additional imaging. LR-4 observations indicate a high probability of HCC but do not establish a definitive diagnosis; therefore, repeat imaging, alternative imaging modalities, biopsy, or multidisciplinary assessment may be appropriate depending on the clinical context. In contrast, LR-5 observations establish a non-invasive imaging diagnosis of HCC in at-risk patients [11,91,106]. The corresponding management recommendations are summarized in the management recommendations section.

Role and diagnostic performance of major features: The major imaging features used in CT/MRI LI-RADS include nonrim APHE, nonperipheral washout, enhancing capsule, and threshold growth [21,142]. The classic imaging appearance of HCC on multiphasic CT and MRI consists of APHE, followed by washout and the presence of an enhancing capsule on portal venous and/or delayed phase imaging [143]. Substantial inter-reader agreement has been reported for both assessment of major imaging features and overall LI-RADS category assignment [144]. Among these features, APHE and washout demonstrate the strongest associations with HCC diagnosis [145]. A systematic review and meta-analysis reported that APHE provides the highest sensitivity, whereas enhancing capsule demonstrates the highest specificity for HCC diagnosis [146]. Consistent with these findings, major imaging features have shown high specificity for diagnosing HCC in 10-19 mm observations demonstrating APHE [147]. Furthermore, incorporation of transitional phase and hepatobiliary phase hypointensity into washout assessment has been shown to improve diagnostic sensitivity, albeit with a modest reduction in specificity [148].

The role of threshold growth remains controversial. Several studies have reported that threshold growth contributes less to HCC diagnosis than other major imaging features and may not represent an independently significant predictor of HCC [145,149]. A recent Korean study further demonstrated that reclassifying threshold growth as an AF did not significantly alter the diagnostic performance of the LR-5 category [150]. Nevertheless, threshold growth remains a major feature within the current CT/MRI LI-RADS algorithm.

Application of ancillary features and tie-breaking rules: AFs may be applied at the discretion of the interpreting radiologist to improve lesion detection, increase diagnostic confidence, and refine category assignment [151]. The presence of one or more AFs favoring malignancy permits upgrading an observation by one category, up to a maximum of LR-4, whereas AFs should never be used to upgrade an observation to LR-5 [20,119]. Conversely, the absence of malignancy-favoring AFs should not be used to justify category downgrading. In situations of diagnostic uncertainty, LI-RADS tie-breaking rules recommend assigning the category associated with the lower degree of certainty. Similarly, when uncertainty exists regarding the presence of TIV, LR-TIV should not be assigned [140].

LR-1 and LR-2 observations: LR-1 (definitely benign) observations represent lesions with imaging features that are unequivocally benign. Typical examples include cysts, hemangiomas, perfusion alterations such as arterioportal shunts, hepatic fat deposition or focal fat sparing, hypertrophic pseudomasses, and areas of confluent fibrosis or focal scar [11,152]. Lesions that demonstrate spontaneous disappearance on follow-up imaging are also categorized as LR-1 [140,153].

LR-2 (probably benign) observations are likely benign but do not show the completely definitive imaging features required for LR-1 classification. These include probable cysts, probable hemangiomas, probable perfusion alterations (e.g., arterioportal shunts), probable focal fat deposition or sparing, probable hypertrophic pseudomasses, and probable confluent fibrosis or focal scars [11]. LR-2 may also include a distinctive nodule, defined as a solid nodule <20 mm that appears different from background nodules but lacks any major imaging feature of HCC, LR-M features, or AFs favoring malignancy. Such nodules may demonstrate T1 hyperintensity, T2 hypointensity, siderotic (iron-containing) appearance, hepatobiliary phase hyperintensity, or combinations of these findings [11]. Lesions measuring ≥20 mm should not be categorized as LR-2 and instead should be assigned LR-3 or higher based on their imaging characteristics [153].

Diagnostic performance of CT/MRI LI-RADS v2018: In a retrospective MRI-based study, LI-RADS v2018 demonstrated higher sensitivity, negative predictive value, and overall diagnostic accuracy for HCC than LI-RADS v2017 [154]. A systematic review reported pooled sensitivity and specificity of 83% and 91%, respectively, for LI-RADS v2018 in the diagnosis of HCC [155]. Similarly, another meta-analysis demonstrated pooled sensitivity and specificity of 0.80 and 0.89, respectively, confirming the robust diagnostic performance of LI-RADS for HCC detection [156].

The diagnostic accuracy of LI-RADS varies according to the category threshold used to define HCC. A meta-analysis showed that LI-RADS ≥3 provides excellent overall diagnostic performance, with pooled sensitivity and specificity of 86% and 85%, respectively. However, increasing the diagnostic threshold results in higher specificity at the expense of sensitivity, with LI-RADS ≥5 demonstrating substantially greater specificity than LI-RADS ≥3 or LI-RADS ≥4 [157].

Several studies have specifically evaluated the performance of the LR-5 category. A systematic review and meta-analysis demonstrated excellent diagnostic performance of MRI LI-RADS v2018 using both LR-5 alone and the combined LR-4/5 categories for evaluating suspected malignant liver nodules [158]. Likewise, a meta-analysis by Lee et al. reported that LR-5 provides moderate sensitivity (70%) but high specificity (91%) for the noninvasive diagnosis of HCC on a per-observation basis [159]. A more recent systematic review published in 2024 further confirmed the consistently high specificity of the LR-5 category across different imaging modalities and contrast agents, while showing that MRI performed with ECAs provides higher sensitivity than CT and gadoxetate-enhanced MRI [160]. These pooled diagnostic performance estimates should be interpreted within the intended LI-RADS target population and should not be generalized to patients without established HCC risk factors.

In addition to its diagnostic accuracy, LI-RADS has demonstrated good reproducibility. Multiple studies have reported moderate-to-substantial inter-reader agreement for both LI-RADS category assignment and assessment of individual imaging features while maintaining high specificity for differentiating HCC from non-HCC primary liver malignancies [161].

CT/MRI LI-RADS-based management - suggested imaging workup options and time intervals: The assigned CT/MRI LI-RADS category not only reflects the estimated probability of HCC and overall malignancy but also provides guidance for subsequent imaging evaluation and clinical management. Management recommendations vary according to the assigned category, ranging from routine surveillance for definitely or probably benign observations to repeat diagnostic imaging, MDD, biopsy, or definitive treatment for observations with increasing suspicion of HCC or other malignancies. However, final management decisions should integrate the LI-RADS category with the overall clinical context and multidisciplinary team discussion to ensure individualized patient care. The suggested CT/MRI-based imaging workup options and recommended follow-up intervals according to LI-RADS v2018 are summarized in Table 14 [105].

**Table 14:** LI-RADS categorization and management for untreated observations. Source: Adapted from the American College of Radiology CT/MRI LI-RADS Diagnostic Algorithm v2018 Core [11]. Abbreviations: LI-RADS, Liver Imaging Reporting and Data System.

LI-RADS category	Definition	Recommended management
LR-NC	Not categorizable	Repeat or perform alternative diagnostic imaging within ≤ 3 months
LR-1	Definitely benign	Return to routine surveillance in 6 months
LR-2	Probably benign	Return to surveillance in 6 months; consider repeat diagnostic imaging within ≤ 6 months
LR-3	Intermediate probability of hepatocellular carcinoma (HCC)	Repeat or alternative diagnostic imaging in 3-6 months
LR-4	Probably HCC	Multidisciplinary discussion for tailored workup; may include biopsy
LR-5	Definitely HCC	HCC confirmed; multidisciplinary discussion for consensus management
LR-M	Probably or definitely malignant but not specific for HCC	Multidisciplinary discussion for tailored workup; often includes biopsy
LR-TIV	Tumor in vein	Multidisciplinary discussion for tailored workup; may include biopsy

LI-RADS treatment response assessment (TRA)

General Principles

Conventional size-based response criteria, such as RECIST 1.1 (Response Evaluation Criteria in Solid Tumors), are inadequate for assessing treatment response in HCC because locoregional therapies frequently result in tumor necrosis without immediate lesion shrinkage. Enhancement-based criteria, including the EASL criteria and modified Response Evaluation Criteria in Solid Tumors (mRECIST), were subsequently developed to evaluate the viable enhancing component of the tumor. Building upon these principles, LI-RADS TRA provides a standardized, lesion-specific framework for imaging-based TRA by categorizing treated observations according to imaging features of tumor viability, thereby facilitating consistent interpretation and reporting following locoregional therapy (LRT) [13].

The LI-RADS Treatment Response Assessment (LR-TR) was first introduced in 2017 and refined in 2024, incorporating evolving evidence and modality-specific considerations. Until the 2024 update, TRA within LI-RADS was performed exclusively using CECT or MRI. A major advancement in LI-RADS v2024 was the incorporation of CEUS into the treatment response framework. The updated algorithm distinguishes between radiation-based and non-radiation-based therapies and provides separate assessment pathways for each. Response to non-radiation-based therapies may now be evaluated using either CEUS or CT/MRI, whereas response to radiation-based therapies continues to be assessed exclusively with CT/MRI. Furthermore, v2024 also clarifies and simplifies assessment of viability after non-radiation-based therapies as well as incorporates AFs, including diffusion restriction and mild-to-moderate T2 hyperintensity, as optional adjuncts for evaluating residual tumor viability [13-15,18,162,163].

The LR-TR v2024 algorithm has demonstrated robust performance for the detection of residual viable tumor following LRT [164]. Emerging evidence also supports the role of CEUS in TRA. A prospective pilot study by McGillen et al. demonstrated that CEUS is comparable to CT/MRI performed at identical follow-up intervals for differentiating residual viable tumor from successfully treated HCC after initial transarterial chemoembolization (TACE) [165]. Similarly, a retrospective observational study by Kuon Yeng Escalante et al. reported that CEUS nonradiation LR-TR provides reliable detection of viable tumor in TACE-treated lesions and demonstrates good inter-reader reproducibility [166].

Nonradiation-Based Treatment Response

Applicable therapies: Nonradiation-based therapies include ablation, TACE, bland embolization, and surgical resection [167-169]. These therapies typically induce immediate devascularization.

Expected imaging appearance after LRT: After nonradiation-based LRT, tumor response is typically immediate and reflects the mechanism of treatment. Following thermal or chemical ablation, such as radiofrequency ablation (RFA), microwave ablation (MWA), or percutaneous ethanol ablation (PEA), cellular injury leads to protein denaturation and coagulative necrosis [167].

In thermal ablation, the ablation zone is intentionally larger than the original tumor, often by about 10 mm to ensure eradication of microscopic disease, whereas in chemical ablation, the treatment zone usually approximates the size of the pre-treatment lesion. Over time, treated lesions are expected to decrease in size. Small intralesional gas foci may be seen shortly after ablation but should resolve within seven to 10 days; persistence or new gas raises concern for abscess formation [168].

After intra-arterial embolic therapies such as transarterial embolization (TAE), cTACE, or DEB-TACE, tumor response also occurs immediately and results from ischemic injury, chemotoxic effects, or both [169]. Echogenic intralesional material, including air or embolic particles, may persist for days to weeks, but persistent or newly developing gas should prompt consideration of infection [167].

Changes in the surrounding liver parenchyma are common after LRT and may include transient perfusional or inflammatory alterations, often appearing as a smooth rim of arterial hyperenhancement without washout around the treated lesion. Wedge-shaped or geographic hypoenhancing areas related to parenchymal injury may also be observed, and a linear hyperenhancing tract extending toward the liver capsule can occur after percutaneous ablation due to needle-tract effects. These peri-treatment changes typically resolve within three to six months [170].

LI-RADS CEUS Nonradiation Treatment Response Algorithm (TRA) v2024

Indications and contraindications: The LI-RADS CEUS Nonradiation Treatment Response Algorithm (TRA) v2024 is intended for use in high-risk patients to assess treatment response of pathologically proven or presumed HCC, including observations previously categorized as LR-3, LR-4, LR-5, or LR-M, after LRT [15]. High-risk patients include those with cirrhosis, chronic hepatitis B infection (with or without cirrhosis), or current or prior HCC, including adult liver transplant candidates and recipients [15]. This algorithm should be applied only to treated lesions after nonradiation-based locoregional therapies such as RFA, MWA, PEA, TAE, cTACE, or DEB-TACE [15,162,171]. It should be applied only when the treated lesion is visible on post-treatment B-mode ultrasound and may be used in postsurgical patients to evaluate for recurrence at the surgical margin if the cavity or margin is visible on ultrasound. In selected cases, cautious application may be considered for certain non-HCC malignancies, such as intrahepatic cholangiocarcinoma (iCCA) and combined hepatocellular-cholangiocarcinoma (cHCC-CCA) [15,172].

The algorithm should not be applied when the treated lesion is not visible on B-mode ultrasound, when evaluating new or untreated lesions outside the treatment zone, in patients who have undergone radiation-based therapies, or in patients receiving systemic therapy [15].

Technical recommendations: The successful application of the LI-RADS CEUS Nonradiation Treatment Response Algorithm (TRA) v2024 requires adherence to standardized technical principles to ensure accurate evaluation of treated liver lesions. Appropriate examiner training is essential. Operators and readers should be proficient in liver CEUS technique, comprehensive documentation of all enhancement phases, correct contrast agent administration, optimization of imaging parameters, artifact reduction, and management of rare contrast-related adverse reactions [15].

CEUS examinations should conform to the technical guidance provided by the ACR CEUS LI-RADS Working Group and the American College of Radiology/American Institute of Ultrasound in Medicine/Society of Radiologists in Ultrasound (ACR-AIUM-SRU) Practice Parameter for CEUS performance [45].

Ultrasound equipment must be equipped with CEUS-specific software and hardware capabilities. A low-frequency curved-array transducer is generally preferred for liver imaging, while high-frequency or linear transducers may be used for superficially located lesions when visualization with a curved-array probe is suboptimal [15].

Two purely intravascular microbubble contrast agents are commonly utilized for liver CEUS: Lumason/SonoVue (sulfur hexafluoride lipid-type A microspheres) and Definity/Luminity (perflutren lipid microspheres) [15]. Contrast dose should be individualized according to equipment sensitivity, patient body habitus, and underlying liver condition. Typical dosing for liver applications includes 1.0-2.4 mL for Lumason/SonoVue and 0.2-0.4 mL for Definity/Luminity [15].

Patients are typically positioned supine with the right arm abducted to optimize intercostal access [15]. Following identification of an optimal acoustic window, baseline B-mode imaging is performed, and the treated lesion is measured prior to contrast administration [15]. Contrast is administered through an 18-22-gauge peripheral intravenous catheter; central venous lines or ports may be used in accordance with institutional protocols. Each contrast bolus should be immediately followed by a 5-10 mL normal saline flush [15].

The imaging protocol includes continuous real-time imaging from the time of contrast injection through the peak arterial phase, optionally extending up to 60 seconds, to adequately assess APHE within the treated lesion and its margins. After 60 seconds, intermittent imaging (5-10 seconds every 30-60 seconds) is recommended to evaluate for washout characteristics during the portal and late phases [15].

CEUS nonradiation treatment response algorithm: CEUS enables real-time assessment of tumor viability following LRTs such as ablation and embolization by evaluating residual vascularity within and around treated lesions. The CEUS LI-RADS Treatment Response Algorithm (TRA) provides a standardized framework for determining treatment response based on enhancement patterns while distinguishing viable tumor from expected post-treatment changes, including peripheral reactive hyperemia [15]. Although CEUS is highly sensitive for detecting small foci of residual viable tumor, its diagnostic performance may be limited by inadequate acoustic windows and the inability to evaluate the entire liver in a single examination [15]. A systematic stepwise approach facilitates consistent interpretation and reporting.

Step 1 - Assessment of tumor viability: Evaluation begins with the assessment of both intralesional and perilesional enhancement using established CEUS criteria. When image quality is insufficient for reliable assessment, the lesion should be categorized as LR-TR nonevaluable, and the evaluation proceeds directly to the final verification step.

Intralesional viability is considered absent when no enhancement is identified within the treated lesion. It is considered uncertain when arterial-phase hypoenhancement is present, with or without washout, and present when arterial-phase hyperenhancement or isoenhancement is identified, regardless of washout [15,171].

Perilesional viability is considered absent when enhancement is identical to the surrounding liver parenchyma. It is considered uncertain in the presence of arterial-phase hyperenhancement without washout, arterial-phase isoenhancement with washout, or arterial-phase hypoenhancement. Perilesional viability is considered present only when arterial-phase hyperenhancement is followed by washout [15,171]. After partial hepatectomy, the entire surgical margin should be evaluated using perilesional viability criteria, whereas absence of a visible surgical cavity on B-mode ultrasound is interpreted as absent intralesional viability [15].

Step 2 - Apply the tie-breaking rule: When intralesional and perilesional assessments are discordant or when differentiation between adjacent categories is uncertain, the predefined tie-breaking rule should be applied. If distinction between absent and uncertain or between uncertain and present cannot be made confidently, the lesion should be categorized as uncertain [171].

Different assessment criteria are intentionally applied to intralesional and perilesional enhancement. Broader intralesional criteria maximize sensitivity for detecting residual viable tumor, whereas stricter perilesional criteria reduce false-positive interpretation of reactive post-treatment hyperemia [15,173].

**Figure 6:**
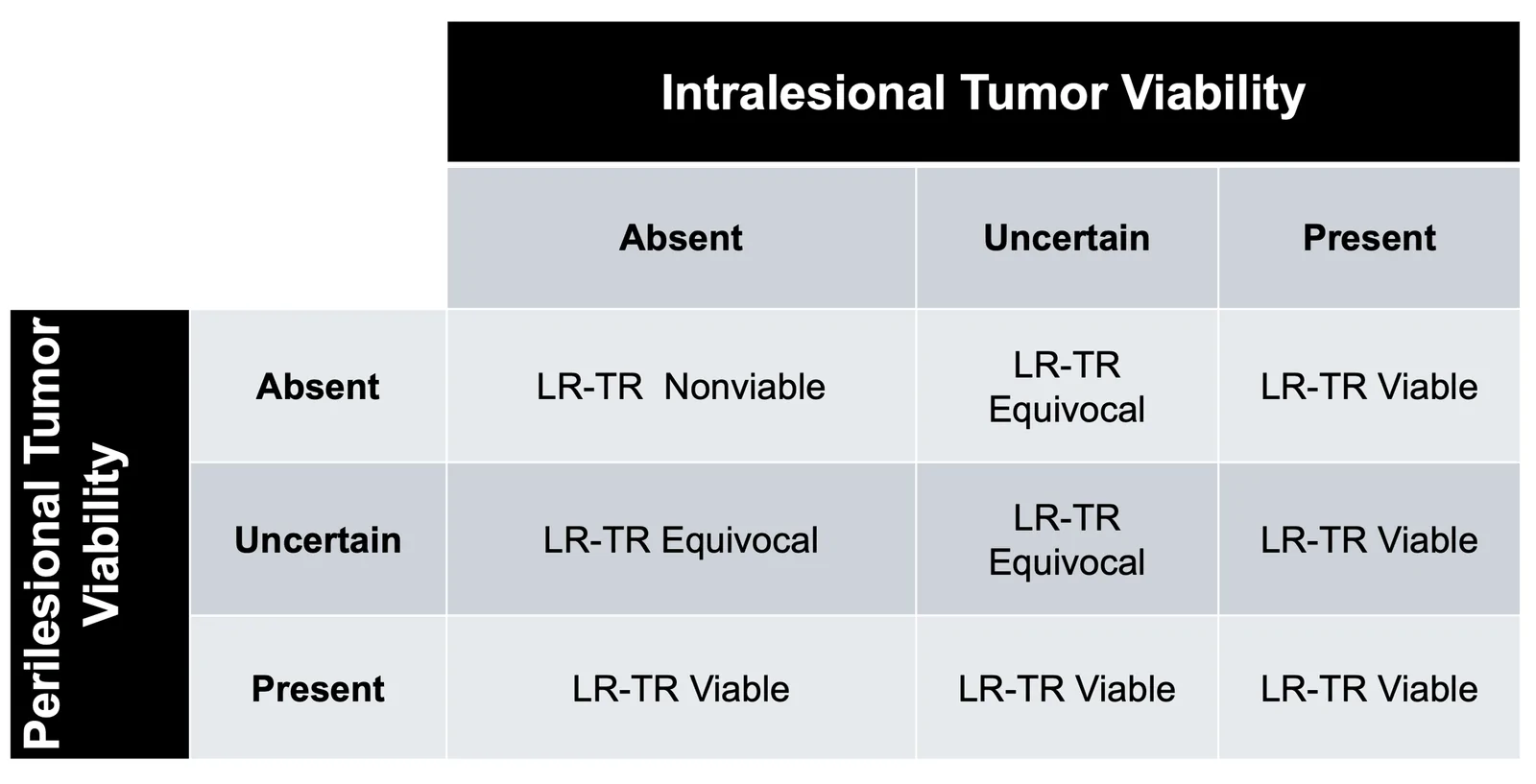
Decision matrix for CEUS LI-RADS Treatment Response Assessment (TRA) category assignment based on intralesional and perilesional tumor viability. Adapted and redrawn by the authors from the American College of Radiology CT/MRI LI-RADS Treatment Response Algorithm v2024 [15].

Step 3 - Assign the treatment response category: The final CEUS TRA category is assigned by integrating intralesional and perilesional viability findings into a single overall assessment. This decision process is summarized in Figure 6 [15].

Step 4 - Final verification: The assigned category should be reviewed to ensure that it accurately reflects the imaging findings before proceeding to evaluation of additional treated lesions, when present [15]. Newly detected hepatic observations separate from the treated lesion should be evaluated using the CEUS diagnostic algorithm rather than the CEUS TRA [15,171,173].

CEUS nonradiation TRA categories: Treatment response is classified into four categories according to the CEUS LI-RADS TRA [15]. (1) LR-TR Nonevaluable: Treatment response cannot be assessed because of image omission or image degradation. (2) LR-TR Nonviable: Probably or definitely nonviable tumor. (3) LR-TR Equivocal: Imaging findings are indeterminate for viable tumor. (4) LR-TR Viable: Probably or definitely viable tumor.

LI-RADS CEUS nonradiation treatment response algorithm (v2024) - Lexicon: To facilitate standardized TRA, LI-RADS v2024 provides a dedicated CEUS treatment response lexicon [15].

Viability refers to the presence of live tumor cells within a treated lesion or along its margin [15]. The definition of a treated lesion depends on the treatment modality. Following catheter-based therapies, including TAE, cTACE, and DEB-TACE, the treated lesion corresponds to the treated observation identified on B-mode ultrasound. After percutaneous therapies such as RFA, MWA, or PEA, it includes both the treated observation and the surrounding ablation zone. Following surgical resection, the treated lesion corresponds to the postoperative surgical cavity visualized on B-mode ultrasound [15].

Intralesional contrast enhancement describes enhancement occurring within the margins of the treated lesion, whereas perilesional contrast enhancement refers to enhancement immediately adjacent to the outer margin of the treated lesion. Following surgical resection, enhancement along the resection margin is regarded as perilesional enhancement [15].

Intralesional tumor viability is determined primarily from arterial-phase imaging. Intralesional arterial-phase hyperenhancement or isoenhancement indicates viable tumor, whereas washout is not essential but increases diagnostic confidence. If no surgical cavity is visible on B-mode ultrasound, intralesional viability is classified as absent [15]. Perilesional tumor viability represents viable tumor along the treated lesion margin and requires arterial-phase hyperenhancement, followed by washout. After partial hepatectomy, the entire resection margin should be assessed using these criteria [15].

Limitations of CEUS LI-RADS Nonradiation TRA v2024: The ACR CEUS LI-RADS TRA for nonradiation therapies has several recognized limitations. An LR-TR Nonviable assessment should not be interpreted as evidence of complete pathologic response because CEUS, like other imaging modalities, may fail to detect microscopic or very small foci of residual viable tumor that are identifiable only on histopathologic examination [171]. Assessment of treatment response may also be challenging in certain atypical HCCs. In biopsy-proven HCCs that demonstrate arterial-phase isoenhancement without washout on pre-treatment CEUS (initially categorized as LR-3), evaluation of perilesional tumor viability following treatment may be difficult, and additional assessment with CT, MRI, or short-interval follow-up imaging may be necessary. Likewise, in the uncommon setting of biopsy-proven HCC demonstrating arterial-phase hypoenhancement before treatment, both intralesional and perilesional viability assessment may be unreliable, making alternative cross-sectional imaging or interval follow-up advisable [171].

Current limitations also extend to treatment type and contrast agent. The CEUS TRA has not yet been validated for assessing response after radiation-based therapies, including TARE and SBRT, or following systemic therapies because of limited available evidence [171]. Similarly, ultrasound contrast agents such as Optison (perflutren protein-type A microspheres) and Sonazoid (perfluorobutane microspheres with Kupffer-phase imaging) are not currently incorporated into the algorithm owing to insufficient validation for TRA in HCC [174].

Management recommendations: The ACR CEUS LI-RADS TRA does not specify a fixed interval for post-treatment CEUS following LRT. Instead, the timing of imaging is determined by the treatment modality, the expected duration of post-procedural reactive changes, institutional protocols, and individual patient factors. Consequently, the choice of imaging modality and follow-up interval should be individualized through MDD [15,172]. General management recommendations according to the CEUS TRA category are summarized in Table 15 [15,172]. Newly detected nodules that are spatially separate from the treated lesion should be evaluated using the CEUS LI-RADS diagnostic algorithm rather than the TRA [15].

**Table 15:** Recommended management for treatment response category. * Follow-up imaging may be performed using the same or an alternative imaging modality as clinically appropriate. † If stable for one to two years, the follow-up interval may be extended to six months. Source: Adapted from the American College of Radiology CEUS LI-RADS Nonradiation Treatment Response Assessment v2024 Core [15]. Abbreviations: CT, computed tomography; MRI, magnetic resonance imaging.

Treatment response category	Clinical scenario	Recommended management
LR-TR Nonevaluable	At any time after treatment	Repeat imaging within 3 months*
LR-TR Nonviable	At any time after treatment	Continue surveillance every 3 months*†
LR-TR Equivocal	< 6 months after treatment, or decreasing areas of uncertain viability ≥ 6 months after treatment	Continue surveillance every 3 months*; consider multidisciplinary discussion (MDD) in unusual or complex cases
	Persistent areas of uncertain viability ≥ 6 months after treatment, or new/increasing areas of uncertain viability	Multidisciplinary discussion for consensus management; often includes CT or MRI
LR-TR Viable	At any time after treatment	Multidisciplinary discussion for consensus management; retreatment is commonly indicated

LI-RADS CT/MRI TRA v2024

What’s new in LI-RADS CT/MRI TRA v2024: The ACR LI-RADS CT/MRI TRA v2024 introduces a major structural revision by dividing the system into two distinct TRA cores based on the type of LRT [175]. The Nonradiation TRA Core is designed for assessment after nonradiation-based therapies, including thermal ablation (e.g., RFA and MWA), chemical ablation (e.g., PEA), and surgical resection. In contrast, the Radiation TRA Core is specifically intended for evaluation following radiation-based therapies such as external beam radiation therapy (EBRT), SBRT, and TARE [13,14,19,176].

This update reflects the recognition that post-treatment imaging appearances differ fundamentally between radiation-based and non-radiation-based therapies. In prior LI-RADS versions, both were assessed using a single unified TRA algorithm. The 2024 revision improves conceptual clarity, diagnostic accuracy, and reporting consistency by tailoring response criteria to the specific biological and imaging effects of each treatment category.

Another key update is the simplification of viability criteria. In earlier versions (e.g., v2017), LR-TR Viable was defined using multiple major features, including nodular, mass-like, or thick irregular tissue demonstrating APHE, washout, or enhancement similar to the pretreatment appearance. In v2024, within the Nonradiation TRA Core, LR-TR Viable is defined by a single feature, mass-like enhancement of any degree in any phase, streamlining interpretation and improving usability [14,39,163].

Additionally, AFs favoring viability have been introduced, allowing optional upgrade (at the user’s discretion) from LR-TR Equivocal or LR-TR Nonprogressing to LR-TR Viable. For the Nonradiation TRA Core, such AFs include diffusion restriction of any degree or mild-to-moderate T2 hyperintensity in areas of uncertain enhancement. These additions enhance confidence in identifying viable tumor while maintaining flexibility in clinical decision-making [14,19].

LI-RADS TRA v2024 enables earlier detection of local tumor recurrence and identifies recurrent lesions at smaller sizes compared with the CT/MRI LI-RADS v2018 diagnostic algorithm, resulting in earlier diagnosis in approximately 28% of patients [173].

Indications and contraindications of CT/MRI TRA: The ACR LI-RADS TRA should be applied in high-risk patients to assess treatment response of pathologically proven or presumed HCC, including observations previously categorized as LR-4, LR-5, or LR-M, following LRT. High-risk populations include individuals with cirrhosis of any etiology, chronic hepatitis B infection with or without cirrhosis, current or prior HCC, adult liver transplant candidates, and post-liver transplant recipients. The algorithm is intended for treated lesions evaluated with post-treatment multiphasic CT or MRI, including studies performed with ECAs or hepatobiliary contrast agents [13,14].

The nonradiation TRA algorithm is applicable after therapies such as RFA, MWA, cryoablation, PEA, TAE, cTACE, and DEB-TACE, and it may also be used in the postsurgical setting when assessing suspected recurrence at a surgical margin [177]. In contrast, the radiation TRA algorithm should be applied following radiation-based therapies such as SBRT or TARE [13,14].

The LI-RADS TRA should not be applied when imaging is limited to noncontrast or single-phase CT or MRI, as these studies are inadequate for TRA. It is also not intended for evaluating new or untreated lesions outside the treatment zone or for assessing observations distant from the surgical margin in postsurgical patients. Furthermore, the algorithm should not be used in patients treated with systemic therapy alone and should be applied cautiously in patients receiving combined systemic and locoregional treatments, as treatment effects may be more complex to interpret [13,14].

Expected imaging appearance after SBRT or TARE: Expected post-treatment imaging changes following SBRT and TARE may overlap with findings suggestive of residual tumor. Familiarity with these treatment-related alterations is essential for accurate TRA and avoidance of false-positive interpretations.

Persistent APHE during the first three months after treatment should not automatically be considered evidence of residual tumor, as it may reflect post-radiation effects. Careful comparison with prior studies is essential, because a progressive decrease in enhancement or lesion size is more indicative of treatment response. Perilesional rim enhancement and geographic perfusion changes are frequent post-radiation findings and should not be mistaken for tumor progression [13]. In cases treated with high-dose segmental radioembolization, early absence of enhancement is expected due to devascularization and should be interpreted as a favorable response rather than a concerning finding [13,168].

After radiation-based LRT, the expected imaging appearance varies according to treatment type, dosimetry, and time since therapy. Following SBRT or TARE performed with standard dosimetry, early imaging within the first three months may show persistent masslike APHE and washout within the treated lesion [170,178]. Smooth perilesional rim enhancement is commonly present, and moderate to significant geographic perfusion changes may be seen in the surrounding liver parenchyma [13]. On later follow-up beyond six months, a gradual decrease in lesion size and APHE is typically observed. A delayed-phase capsule or halo may appear, parenchymal perfusion abnormalities gradually diminish, sometimes shifting from arterial phase to portal venous or delayed phase enhancement, and progressive atrophy of the liver in the treated zone may develop [13].

After TARE performed with segmental or ablative high-dose dosimetry, early imaging within three months often demonstrates no intralesional enhancement because of complete devascularization and necrosis, which is an expected finding. Smooth perilesional rim enhancement and moderate to significant geographic perfusion changes in adjacent liver parenchyma may still be present [13]. On later imaging beyond six months, intralesional enhancement typically remains absent, while perfusion changes gradually decrease and may shift in phase due to fibrosis, and progressive atrophy of the treated liver segment may be seen [13,170].

LI-RADS treatment response features: Assessment of treatment response in LI-RADS is based on imaging features that reflect residual tumor viability following locoregional or surgical therapies. The LI-RADS TRA provides standardized definitions for these features, which are discussed below [13,14].

Viability refers to the presence of living tumor cells within a treated lesion or along its margin. Importantly, radiologic viability is not equivalent to pathologic viability, as imaging modalities lack sufficient sensitivity to detect microscopic or very small foci of residual tumor [13,14]. Accordingly, absence of enhancement should not be equated with complete pathological necrosis, as small foci of residual viable tumor may remain below the detection threshold of imaging. LI-RADS CT/MRI TRA v2024 defines a major feature of viability as an imaging finding that independently warrants assignment of the LR-TR Viable category. In the current version, masslike enhancement is the sole major feature used to establish viability [179]. Masslike enhancement is defined as an enhancing area of any degree, observed on any contrast-enhanced phase, that occupies space. Examples include nodular enhancement, irregular peripheral enhancement, or a thick enhancing rim [13,14,177].

Following non-radiation-based LRT or surgical resection, masslike enhancement within the treated lesion, along its margin, or at the surgical margin is interpreted as viable tumor. If there is uncertainty regarding the presence or morphology of masslike enhancement, the finding should be categorized as equivocal for viable tumor [13,14]. In contrast, after radiation-based LRT, interpretation depends on temporal evolution: new or increasing masslike enhancement over time indicates viable tumor, whereas stable or decreasing masslike enhancement is interpreted as nonprogressing tumor [13,14]. Tumor-marker trends may provide complementary information during follow-up but should not be used in isolation to determine radiological viability.

Ancillary features: The ACR LI-RADS TRA v2024 has introduced the AFs favoring viability, which may be applied at the radiologist’s discretion to upgrade LR-TR Equivocal to LR-TR Viable or LR-TR Nonprogressing to LR-TR Viable [13,14]. There are two such AFs: diffusion restriction (of any degree) and mild-to-moderate T2 hyperintensity [177]. These features apply only to MRI; there are no AFs favoring viability on CT and none favoring nonviability [180]. A systematic review and meta-analysis supports the inclusion of restricted diffusion as an ancillary criterion in the LI-RADS TRA v2024 for radiation-based treatment [181].

Diffusion restriction is defined as signal intensity higher than the liver on diffusion-weighted imaging that cannot be attributed solely to T2 shine-through [13,14]. Inclusion of diffusion-weighted imaging as an ancillary criterion in LI-RADS TRA 2024 facilitates viable tumor identification when enhancement-based assessment is inconclusive, although its limitations after LRT should be considered [182].

Mild-to-moderate T2 hyperintensity refers to signal intensity on T2-weighted imaging that is greater than liver parenchyma, similar to or lower than that of a non-iron-overloaded spleen, and lower than that of simple fluid [13,14]. After non-radiation-based LRT or surgical resection, the presence of one or both AFs, diffusion restriction or mild-to-moderate T2 hyperintensity, within an area of uncertain masslike enhancement may justify upgrading the category from LR-TR Equivocal to LR-TR Viable [13,14]. After radiation-based LRT, these AFs may also be used to upgrade a lesion from LR-TR Nonprogressing to LR-TR Viable if they are new or increasing over time in an area where masslike enhancement is stable or decreasing [13,14].

CT/MRI treatment response categories: LI-RADS TRA v2024 includes distinct treatment response algorithms for radiation-based and non-radiation-based therapies. Both algorithms comprise four treatment response categories, three of which are shared: LR-TR Nonevaluable, LR-TR Nonviable, and LR-TR Viable [13,14]. The non-radiation-based algorithm includes the LR-TR Equivocal category for observations with indeterminate viability, whereas the radiation-based algorithm incorporates the newly introduced LR-TR Nonprogressing category to account for the expected temporal evolution of enhancement following radiation therapy [13,14]. The following sections review each category along with the recommended management approach.

LR-TR Nonevaluable: LR-TR Nonevaluable is assigned when treatment response cannot be meaningfully evaluated because of inappropriate imaging technique or inadequate image quality [170]. The defining criterion is the inability to assess the presence or absence of masslike enhancement due to image degradation or omission of required imaging components. Common causes include motion or other artifacts, absence of one or more required contrast-enhanced phases, contrast injection failure, or gross arterial phase mistiming [13,14,179]. This category should not be applied when all recommended contrast phases have been acquired with acceptable image quality, including proper late arterial phase timing, even if interpretation is challenging because of unusual or atypical imaging features; in such cases, another evaluable TRA category should be selected.

When a lesion is categorized as LR-TR Nonevaluable, repeat post-treatment imaging within three months or sooner is recommended [170]. Repeating the study with the same modality is appropriate when the limitation is due to a correctable technical issue, while use of an alternative modality or contrast agent may be considered if it is expected to improve diagnostic confidence [13,14]. LR-TR Nonevaluable represents a technical limitation rather than an indeterminate biologic response and should prompt timely repeat imaging rather than delayed routine surveillance [13,14].

**Figure 7:**
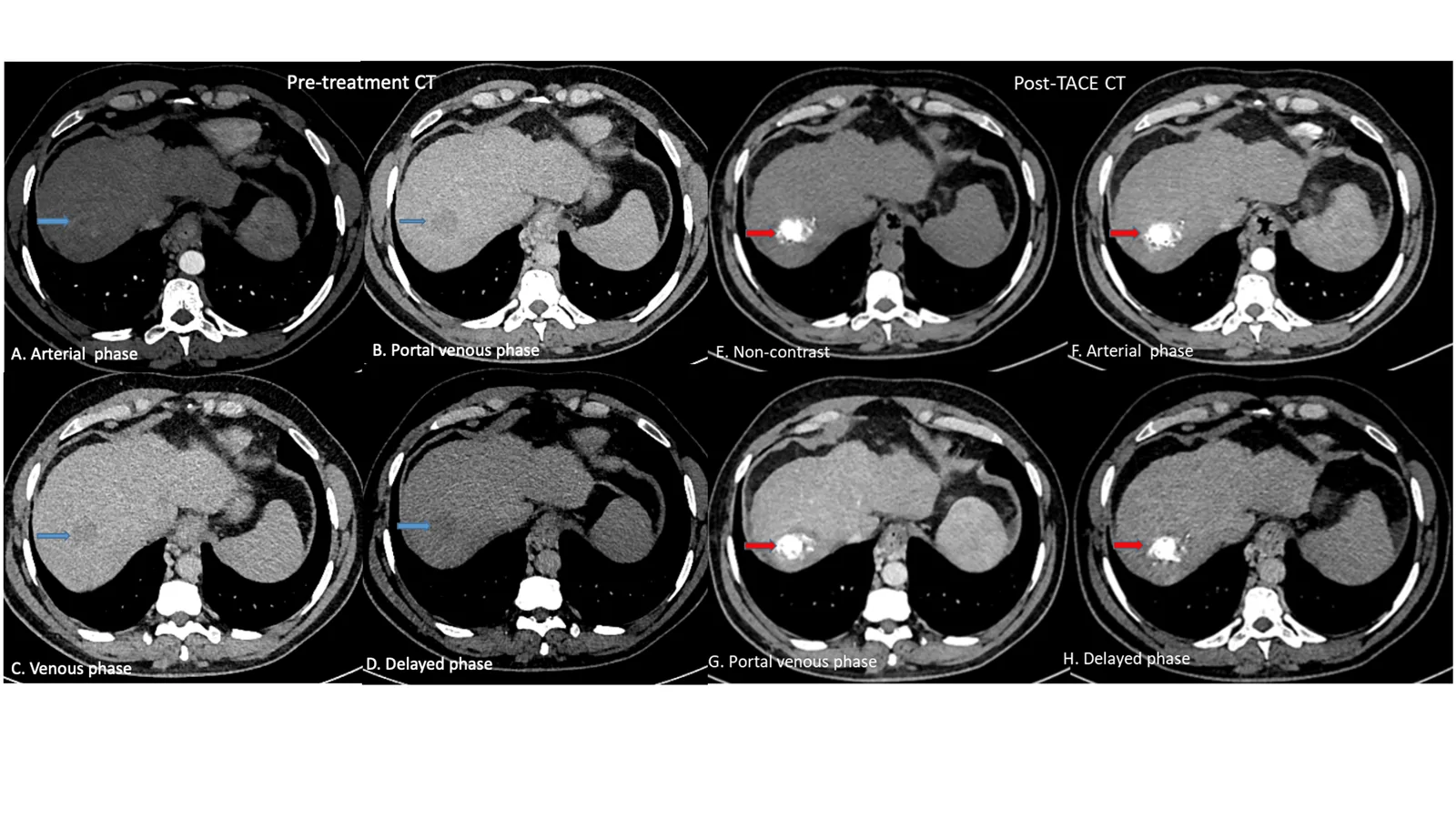
LR-TR nonviable category after conventional TACE on multiphasic CT. Pretreatment axial multiphasic contrast-enhanced CT images (A-D) obtained during the arterial (A), portal venous (B), venous (C), and delayed (D) phases demonstrate a well-defined 17-mm hepatic observation in segment VIII (blue arrow). The observation demonstrates nonrim arterial phase hyperenhancement (A) and nonperipheral washout on the portal venous, venous, and delayed phases (B-D), consistent with an LR-5 observation. Post-treatment axial non-contrast and multiphasic contrast-enhanced CT images (E-H) obtained after conventional transarterial chemoembolization (TACE) during the non-contrast (E), arterial (F), portal venous (G), and delayed (H) phases demonstrate dense lipiodol deposition at the treatment site (red arrow) without nodular, mass-like, or thick irregular arterial phase hyperenhancement within or along the margins of the treated observation, consistent with an LR-TR Nonviable category.

LR-TR Nonviable: The LR-TR Nonviable category indicates a low likelihood of clinically significant viable tumor following treatment. After nonradiation-based LRT or at the surgical margin following resection, this category is assigned when there is no evidence of masslike enhancement within the treated lesion, along its margin, or at the surgical margin [13,14,170]. An illustrative example is shown in Figure 7. Similarly, after radiation-based LRT, the absence of masslike enhancement within the treated lesion or along its margin supports this classification. Typical imaging appearances include complete lesion disappearance, absence of intralesional enhancement, smooth perilesional enhancement, or parenchymal perfusional alterations without associated masslike enhancement. In cases of uncertainty between LR-TR Nonviable and LR-TR Equivocal, the lesion should be categorized as LR-TR Equivocal, whereas uncertainty between LR-TR Nonviable and LR-TR Nonprogressing favors LR-TR Nonprogressing [13,14].

Management generally consists of continued post-treatment surveillance with the same imaging modality at approximately three-month intervals, although the use of an alternative modality or contrast agent may be considered if it provides additional diagnostic value. MDD is recommended in complex or atypical cases [13,14,170,179].

LR-TR Equivocal: The LR-TR Equivocal category reflects an uncertain likelihood of clinically significant viable tumor following treatment. It is assigned when there is ambiguity regarding the presence or morphology of masslike enhancement within the treated lesion, along its margin, or at the surgical margin [14]. This category applies exclusively to the nonradiation treatment response algorithm, including lesions treated with thermal ablation (such as RFA, MWA, or cryoablation), chemical ablation (e.g., percutaneous ethanol injection), intra-arterial therapies (such as TAE, cTACE, or DEB-TACE), or at surgical margins after resection [14]. When differentiation between LR-TR Equivocal and either LR-TR Nonviable or LR-TR Viable is uncertain, the lesion should be categorized as LR-TR Equivocal [14].

Management typically involves repeat imaging with the same modality at approximately three months, which is the preferred approach, although alternative imaging techniques may be employed if they are expected to improve lesion characterization [162]. MDD may be warranted in selected or challenging scenarios [14,179].

**Figure 8:**
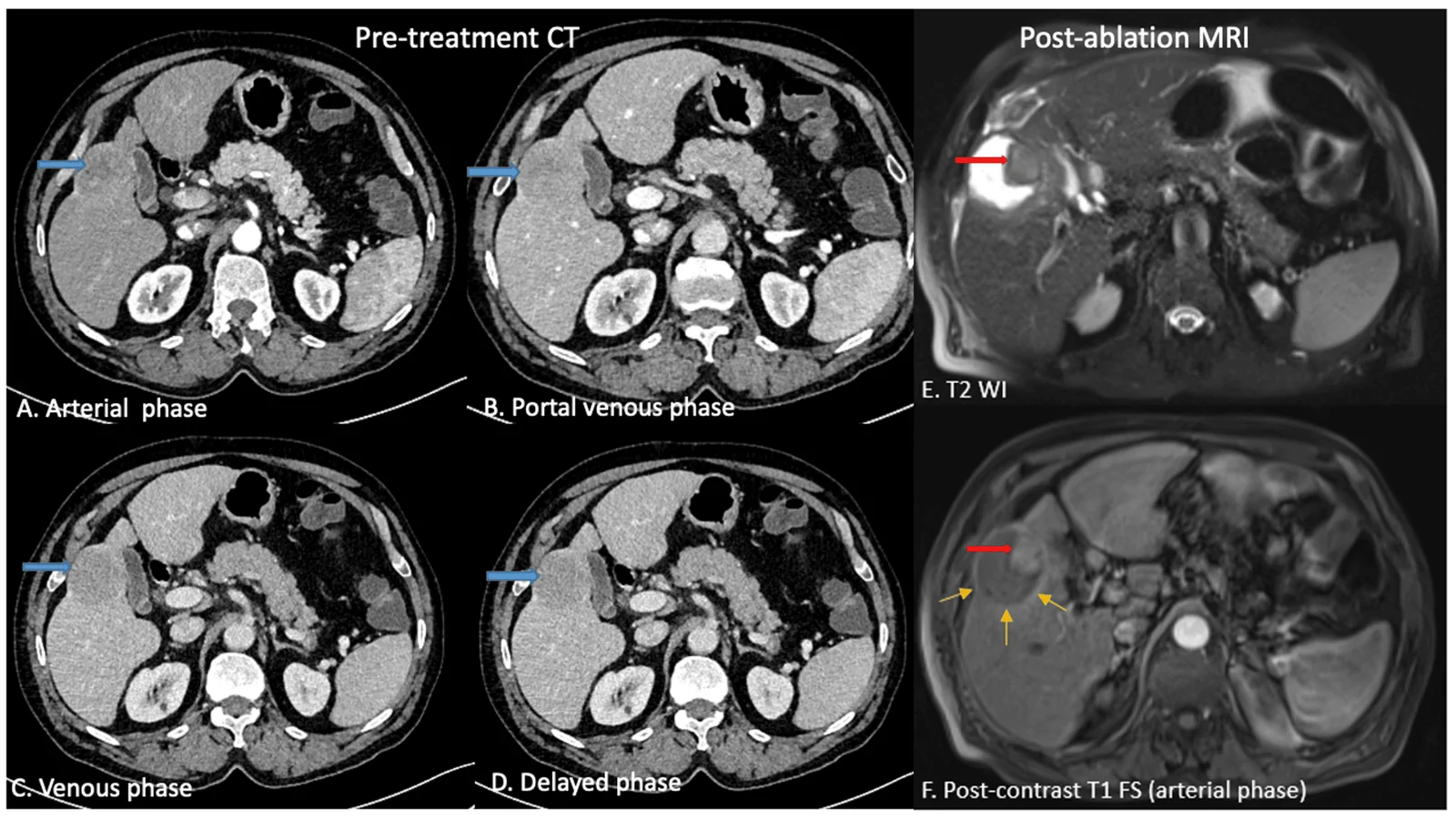
Pretreatment contrast-enhanced CT and post-microwave ablation contrast-enhanced MRI demonstrating an LR-TR Viable observation. Pretreatment axial multiphasic contrast-enhanced CT images obtained during the arterial (A), portal venous (B), venous (C), and delayed (D) phases demonstrate a well-defined hepatic observation measuring >2 cm in segment V (blue arrow). The observation demonstrates nonrim arterial phase hyperenhancement, nonperipheral washout, and an enhancing capsule, consistent with an LR-5 observation. Post-treatment axial MRI images obtained after microwave ablation, including T2-weighted (E) and post-contrast arterial phase T1-weighted fat-saturated (F) images, demonstrate a treated observation with a nodular mass-like T2 hyperintense focus showing nodular mass-like arterial phase hyperenhancement (blue arrow), consistent with an LR-TR Viable category. A smooth, thin peripheral rim of enhancement surrounding the ablation zone (yellow arrow) represents an expected post-ablation imaging finding and should not be mistaken for viable tumor.

LR-TR Viable: The LR-TR Viable category denotes a high likelihood of clinically significant viable tumor after treatment. Following nonradiation-based LRT or at the surgical margin after resection, this category is assigned when there is masslike enhancement of any degree or phase within the treated lesion, along its margin, or at the surgical margin [13,14]. An example of the LR-TR Viable category is illustrated in Figure 8. It may also be applied in cases of indeterminate masslike enhancement when accompanied by AFs such as mild-to-moderate T2 hyperintensity or diffusion restriction. After radiation-based LRT, LR-TR Viable is assigned when there is new or increasing masslike enhancement over time within the treated lesion or along its margin, or when stable or decreasing enhancement is associated with T2 hyperintensity or diffusion restriction. Masslike enhancement may be nodular, smooth, or irregular in morphology [13,14]. In cases of uncertainty between LR-TR Viable and LR-TR Equivocal, the lesion should be categorized as LR-TR Equivocal, while uncertainty between LR-TR Viable and LR-TR Nonprogressing favors LR-TR Nonprogressing [13,14]. Management typically involves MDD for consensus decision-making and often includes consideration of retreatment strategies [14,162,179].

LR-TR Nonprogressing: The LR-TR Nonprogressing category indicates that tumor growth has been effectively arrested following radiation-based LRT, with the expectation that the lesion will eventually evolve toward nonviability. This category is assigned when masslike enhancement, of any degree and in any phase, is present within the treated lesion or along its margin but remains stable or decreases in size over time after treatment [13]. It applies exclusively to the radiation-based treatment response algorithm, including lesions treated with modalities such as SBRT or TARE. In situations of uncertainty between LR-TR Nonprogressing and LR-TR Nonviable or LR-TR Viable, the lesion should be categorized as LR-TR Nonprogressing [13].

Management typically involves continued post-treatment surveillance with the same imaging modality at approximately three-month intervals, which is preferred in most cases [162]. Alternatively, follow-up with a different imaging modality or contrast agent may be considered if it is expected to provide additional diagnostic benefit. MDD is recommended in unusual or complex cases [13,179].

Early post-update evidence has provided preliminary support for the revised radiation-based treatment response categories. In a retrospective study of 242 patients with 319 HCCs treated with ⁹⁰Y radioembolization, v2024 substantially reduced the proportion of observations categorized as LR-TR Viable compared with v2018 (37.3% vs. 87.8%) and classified 57.1% as LR-TR Nonprogressing. All LR-TR Equivocal and 57.5% of LR-TR Viable observations under v2018 were reclassified as LR-TR Nonprogressing with v2024. On subsequent imaging, most observations initially categorized as LR-TR Nonprogressing and LR-TR Viable remained in the same categories, and LR-TR Viable was associated with significantly worse overall survival than LR-TR Nonprogressing, providing early outcome-based support for the revised radiation-specific categories [183].

CT/MRI LI-RADS (TRA) v2024 stepwise approach: The LI-RADS TRA v2024 decision framework provides a stepwise approach for evaluating treated observations and assigning treatment response categories in both non-radiation-based and radiation-based therapy pathways [13,14].

Step 1 - Confirm imaging adequacy: Evaluation of treatment response should begin by determining whether the post-treatment study is technically adequate. Multiphasic CT or MRI must include all required phases and be of sufficient image quality to assess enhancement. If image quality is inadequate or required phases are missing, treatment response cannot be assessed, and the lesion should be categorized as LR-TR Nonevaluable. In such cases, repeat imaging within approximately three months is recommended, preferably using the same modality if the technical limitation is correctable, or an alternative modality if it is expected to provide a diagnostic advantage. If the examination is adequate, assessment proceeds to pathway selection [13,14].

Step 2 - Select the appropriate treatment pathway: (A) Nonradiation pathway: This pathway applies to lesions treated with thermal ablation, chemical ablation, intra-arterial embolization, or observations evaluated at a surgical margin after resection. The key determinant is the presence and characterization of masslike enhancement [14].

If no masslike enhancement is present, the lesion is categorized as LR-TR Nonviable. Findings supporting this category include complete disappearance of the lesion, absence of intralesional enhancement, smooth perilesional enhancement, or parenchymal perfusional changes that do not form a discrete enhancing mass [14]. If masslike enhancement is suspected but its presence or morphology is uncertain, MRI AFs should be assessed. The presence of diffusion restriction or mild-to-moderate T2 hyperintensity in the corresponding area supports viability and allows categorization as LR-TR Viable. If these AFs are absent, the lesion should be categorized as LR-TR Equivocal [14]. If definite masslike enhancement is identified, whether nodular, irregular, or forming a thick enhancing rim, the lesion is categorized as LR-TR Viable [14].

(B) Radiation pathway: This pathway applies to lesions treated with radiation-based therapies such as SBRT or TARE. In this setting, temporal change in enhancement is critical [13].

If no masslike enhancement is present, the lesion is categorized as LR-TR Nonviable [13]. If masslike enhancement is present, prior studies should be reviewed to determine interval change. New or increasing masslike enhancement indicates LR-TR Viable. If enhancement is stable or decreasing, ancillary MRI features should be assessed. Newly appearing or increasing diffusion restriction or mild-to-moderate T2 hyperintensity supports categorization as LR-TR Viable. In the absence of these AFs, the lesion is categorized as LR-TR Nonprogressing [13].

Step 3 - Apply tie-breaker principles: When imaging findings overlap between categories, standardized tie-breaker rules help ensure consistency. If uncertainty exists between Viable and Equivocal, the lesion should be assigned Equivocal. If uncertainty exists between Viable and Nonprogressing, Nonprogressing should be assigned. Similarly, uncertainty between Nonviable and Equivocal favors Equivocal, and uncertainty between Nonviable and Nonprogressing favors Nonprogressing [13,14].

Step 4 - Management overview: Management recommendations depend on the assigned category. LR-TR Nonevaluable requires repeat imaging within approximately three months. LR-TR Nonviable and LR-TR Nonprogressing generally warrant routine post-treatment surveillance at short intervals, typically around three months. LR-TR Equivocal usually prompts short-interval follow-up imaging and, when appropriate, MDD. LR-TR Viable should be discussed in a multidisciplinary setting, and management often includes consideration of retreatment depending on clinical context [13,14].

Limitations

This narrative review provides a comprehensive qualitative synthesis of the evolution, current algorithms, and diagnostic performance of LI-RADS across ultrasound, CEUS, CT/MRI, and TRA. Nevertheless, several limitations of this review should be acknowledged. First, the review primarily focuses on the development, application, and diagnostic performance of LI-RADS and does not provide an in-depth discussion of diagnostic pitfalls encountered in clinical practice, including atypical imaging appearances of HCC, infiltrative tumors, post-treatment imaging changes, and technical factors that may affect lesion characterization and category assignment. Second, emerging modifications of the LI-RADS framework, such as modified LI-RADS (mLI-RADS) and revised LI-RADS (rLI-RADS), which have been proposed to improve diagnostic performance and simplify algorithm application in selected clinical settings, were beyond the scope of this review.

The evidence underlying LI-RADS diagnostic and treatment-response performance is heterogeneous and predominantly derived from retrospective, lesion-based studies. Across studies, reference standards differed according to study design, with histopathologic assessment used in some cohorts and longitudinal imaging or clinical follow-up used in others. Such variation, together with differences in study populations and lesion characteristics, may contribute to spectrum and verification bias. Accordingly, the available evidence primarily supports diagnostic associations, category performance, and reproducibility rather than definitive improvements in patient management or survival outcomes. Guideline recommendations should therefore be distinguished from empirical validation findings, and clinical conclusions should be interpreted in proportion to the strength and design of the supporting evidence.

Finally, although a structured literature search and predefined selection criteria were used, the search was primarily based on PubMed, with additional relevant references identified through cross-referencing. Consequently, relevant studies indexed exclusively in other databases may not have been captured. In addition, formal study quality or risk-of-bias assessment was not performed. As a narrative review, the article is inherently subject to selection bias and does not provide the quantitative synthesis of evidence achievable through a systematic review or meta-analysis.

Future directions

LI-RADS continues to evolve through periodic updates designed to incorporate emerging evidence, technological advances, and user feedback. Future refinements are expected to address changing epidemiological patterns of chronic liver disease and HCC, particularly the increasing burden of MASLD-related HCC, including cases arising in the absence of cirrhosis. Such shifts may necessitate expansion of the current LI-RADS diagnostic population and further validation of the algorithm in patient groups that are currently excluded. Furthermore, given the growing body of evidence supporting the diagnostic performance of Sonazoid-enhanced CEUS for HCC detection and characterization, future versions of CEUS LI-RADS may consider incorporating Sonazoid-specific criteria. However, additional multicenter studies and prospective validation are required before its integration into the LI-RADS framework can be considered. Future integration of artificial intelligence and deep learning into LI-RADS may facilitate automated lesion detection, longitudinal tracking of observations, TRA, and standardized category assignment, potentially improving the accuracy, consistency, and efficiency of liver imaging interpretation.

Molecular imaging represents another potential direction for the future evolution of liver cancer imaging. By providing complementary information on tumor metabolism, differentiation, biological aggressiveness, and tumor burden, molecular imaging may offer information beyond conventional morphologic and vascular assessment. In HCC, approaches including ¹⁸F-fluorodeoxyglucose (FDG) positron emission tomography (PET), dual-tracer PET, and fibroblast activation protein inhibitor (FAPI)-based imaging have shown promising diagnostic and biological applications, with emerging tracers such as Gallium-68-labeled Lannacheng-1007 (⁶⁸Ga-LNC1007) further expanding this field. Although molecular imaging is not currently incorporated into LI-RADS, future studies may determine whether validated molecular imaging biomarkers can complement standardized LI-RADS assessment for tumor characterization, prognostication, and treatment planning. Multicenter prospective validation will be required before their potential integration into the LI-RADS system can be considered [184].

## Conclusions

The LI-RADS has become an important standardized system for liver imaging in patients at risk for HCC. Through successive updates, it has evolved into a comprehensive system encompassing surveillance, diagnosis, and TRA across multiple imaging modalities. The 2024 updates further enhance its clinical utility by refining surveillance recommendations with AFP-guided diagnostic pathways and updated management of subthreshold observations and limited-quality ultrasound examinations, while simplifying TRA through separate algorithms for radiation- and nonradiation-based therapies and the incorporation of CEUS for evaluating nonradiation-based treatments. Continuous familiarity with the LI-RADS lexicon, technical recommendations, and evolving algorithms remains essential for accurate image interpretation, standardized reporting, and multidisciplinary patient management. As the system continues to evolve with emerging evidence and technological advances, LI-RADS is expected to further improve the consistency and clinical applicability of liver imaging.
